# Saracatinib prompts hemin-induced K562 erythroid differentiation but suppresses erythropoiesis of hematopoietic stem cells

**DOI:** 10.1007/s13577-024-01034-5

**Published:** 2024-02-22

**Authors:** Lina Ding, Diyu Chen, Yuanshuai Li, Yingjun Xie, Xiaofang Sun, Ding Wang

**Affiliations:** 1 Department of Obstetrics, Dongguan Songshan Lake Central Hospital, Dongguan Third People’s Hospital, Dongguan, 523326 Guangdong China; 2 Department of Obstetrics and Gynecology, Guangdong Provincial Key Laboratory of Major Obstetric Diseases, The Third Affiliated Hospital of Guangzhou Medical University, No. 63 Duobao Road, Guangzhou, 510150 Guangdong China; 3 Key Laboratory of Reproduction and Genetics of Guangdong Higher Education Institutes, The Third Affiliated Hospital of Guangzhou Medical University, No. 63 Duobao Road, Guangzhou, 510150 Guangdong China

**Keywords:** K562 cells, Hematopoietic stem cells, Erythroid differentiation, Serum starvation, Saracatinib

## Abstract

Human myeloid leukemia cells (such as K562) could be used for the study of erythropoiesis, and mature erythroid markers and globins could be induced during leukemia cell differentiation; however, the pathways involved are different compared with those of hematopoietic stem cells (HSCs).

We identified the differentially expressed genes (DEGs) of K562 cells and HSCs associated with stem cells and erythroid differentiation. Furthermore, we showed that hemin-induced differentiation of K562 cells could be induced by serum starvation or treatment with the tyrosine kinase inhibitor saracatinib. However, erythroid differentiation of HSCs was inhibited by the deprivation of the important serum component erythropoietin (EPO) or treatment with saracatinib. Finally, we found that the mRNA expression of K562 cells and HSCs was different during saracatinib-treated erythroid differentiation, and the DEGs of K562 cells and HSCs associated with tyrosine-protein kinase were identified.

These findings elucidated the cellular phenomenon of saracatinib induction during erythroid differentiation of K562 cells and HSCs, and the potential mechanism is the different mRNA expression profile of tyrosine-protein kinase in K562 cells and HSCs.

**Supplementary Information:**

The online version contains supplementary material available at 10.1007/s13577-024-01034-5.

## Introduction

The study of human erythropoiesis was crucial to understanding and developing treatments for the genetic hemoglobinopathies, including sickle cell disease (SCD) and thalassaemia. Human erythroid differentiation is precisely spatially and temporally regulated. The functional protein of erythroid cells is hemoglobin, including embryonic, fetal and adult proteins, which are formed by two α-clusters and two β-clusters. The embryonic hemoglobins are Hb Gower-1 (ζ2ε2), Hb Gower-2 (α2ε2), Hb Portland (ζ2γ2), and ε-globin (HBE), which are silenced during fetal development [1, 2]. Fetal hemoglobin (HbF) is formed by α-globin and γ-globin (α2γ2) and generated in the yolk sac and the fetal liver; the main adult hemoglobin (α2β2) is formed by α-globin and β-globin, and generated in bone marrow [1, 2]. The transformation of β-globin (HBB) expression to γ-globin (HBG) is an important component of the adaptation to postpartum physiologic and pathologic changes, called hereditary persistence of fetal hemoglobin (HPFH) [2]. The induction of HbF can relieve anemia symptoms in adults and is considered a therapeutic strategy for β-thalassemia patients [3], while the erythroid differentiation of K562 cells is an appropriate cell model [4, 5]. Furthermore, a specific study is needed to explain the difference in drug treatment and signaling pathways between K562 cells and hematopoietic stem cells (HSCs).

There is a close correspondence between cell death and differentiation. Cell death and differentiation are accompanied by biologic phenomena during drug treatment [6]; however, some cell death inducers promote or inhibit cell differentiation [7]. The primary aim of our study was to interpret the mechanism by which autophagy affects erythropoiesis. Normal hematopoietic cells execute erythropoiesis during individual physiologic activity. To maintain physiologic homeostasis, HSCs need to self-renew and maintain pluripotency, and some autophagy-associated proteins are essential in this process [8, 9]. For erythropoiesis, HSCs proliferate and commit early erythroid progenitors; then, the cells mature via terminal erythroid differentiation, and autophagy executes the digestion of redundant organelles and nutrient recovery [10]. An animal study revealed that autophagy-related gene deletion causes anemia because impaired autophagy induces defective erythrocyte maturation [11, 12]. For malignant hematopoietic K562 cells, multivesicular bodies and autophagy have been observed during erythrocyte maturation [13] or differentiation [14, 15]; furthermore, hemotherapeutic drugs induce K562 cell autophagy and increase the expression of erythroid markers [16], and enhancing the differentiation of malignant cancer cells is regarded as an effective anticancer treatment strategy [17].

In the current study, we identified the difference between K562 cells and HSCs in the mRNA expression profile and erythroid differentiation under autophagy inducer treatment. First, a bioinformatics study was conducted to elucidate the difference in K562 cells and HSCs in terms of ‘stem cell’ and ‘erythroid differentiation’. Then, K562 cells were used to study the biologic effect of autophagy on erythroid differentiation. We found that serum-starvation prompted K562 erythroid differentiation and identified that saracatinib effectively promoted erythropoiesis in K562 cells by screening autophagy inducers (rapamycin, vemurafenib and saracatinib). Furthermore, we tested whether serum component deprivation or saracatinib enhanced HSC erythroid differentiation and found that EPO deprivation or saracatinib impaired proliferation during the initial erythropoiesis-induced differentiation of HSCs. Finally, we assessed the mRNA expression of K562 and HSCs in erythropoiesis induction and found some inconsistent results. Based on the saracatinib target, we identified the differentially expressed genes (DEGs) of K562 cells and HSCs as ‘tyrosine-protein kinases’, which may be the potential mechanism explaining the differences in K562 cell and HSC erythroid differentiation in this study. Collectively, we demonstrated that serum starvation and saracatinib enhanced K562 cells, but not HSCs, erythroid differentiation and found that the difference in K562 cells and HSCs in the mRNA expression of reference genes related to erythropoiesis-induced ‘tyrosine-protein kinase’, which prompted the drug target tyrosine-protein kinase to have different biologic effects in the anticancer activity of malignant hematopoietic cells and inhibit erythropoiesis of normal hematopoietic cells.

## Materials and methods

### Clinical materials and ethical approval

Umbilical cord blood was obtained from normal delivery puerpera who signed the informed consent form. The usage of those clinical materials was reported to the Academic Committee of the Third Affiliated Hospital of Guangzhou Medical University and was approved before this study started (No. 2019008), and all experiments were performed in accordance with relevant guidelines and regulations.

### Isolation of hematopoietic stem cells

Umbilical cord blood was diluted 1:1 with PBS and layered on Ficoll. After density gradient centrifugation, the mononuclear cells in the middle layer were collected and then washed twice with PBS. Human CD34 + cells were enriched from the mononuclear cells using CD34 magnetic beads (Cat# 130-100-453, Miltenyi Biotec, Shanghai, China) and stored in liquid nitrogen until use.

### Reagents

Saracatinib (Cat# S1006), vemurafenib (Cat# S1267), rapamycin (Cat# S1039) and hemin (Cat# S5645) were purchased from Selleck Chemicals (Shanghai, China). Recombinant human SCF (Cat# 255-SC-200) was purchased from R&D Systems (Shanghai, China). Recombinant human IL-3 (Cat# GMP200-03) was purchased from Peprotech (Suzhou, China). Erythropoietin (EPO) was purchased from Darbepoetin alfa Administration (Kyowa Hakko Kirin, Japan). StemSpan SFEM (Cat# 09650) was purchased from STEMCELL Technologies. Propylhydrazine hydrochloride (Cat# 114,715) was purchased from Sigma Aldrich.

### Cell culture

K562 cells were gifts from Professor Daolin Tang and were cultured in 1640 medium supplemented with 10% fetal bovine serum (FBS) (Cat# 10,099,141, Gibco, Thermo Fisher Scientific, California, USA), 100 U/ml penicillin and 100 μg/ml streptomycin at 37 °C in 5% CO_2_. For serum-starvation conditions, serum (10% FBS) supplemented 1640 medium was replaced with 2% FBS or serum-free (0% FBS) medium. The cell morphology was recorded using a microscope (TS100, Nikon, Tokyo, Japan).

### Erythroid differentiation

2.5 × 10^5^ per ml K562 cells were committed to differentiation by 60 μM hemin induction for 24 to 72 h. Human CD34 + cells were thawed and induced to differentiate in SFEM for two phases. In the first phase (Days 0–6), media was supplemented with 10% FBS, 50 ng/mL SCF, 10 ng/mL IL-3, and 1 U/mL EPO. In the second phase (Days 7–14), 30% FBS [18, 19] and 3 U/mL EPO were used as supplements.

### Cell-viability assay

K562 cells were treated with the different drugs in 96-well plates according to time- or dose-dependent protocols. After the drug treatments, medium from a Cell Counting Kit-8 (CCK8) (Cat#CK04-3000 T, Dojindo Laboratories, Japan) was added to determine the cell viability by detecting the absorbance at 450 nm, and three to four repeats were used for statistical analysis.

### Reverse transcription-polymerase chain reaction (RT-PCR) analysis

Cells of different groups were lysed using TRIzol (Cat# 12,183,555, Invitrogen, Carlsbad, CA, USA). Subsequently, total RNA was extracted by chloroform and precipitated with isopropanol. The PrimeScript RT Reagent Kit (Cat# RR047A, Takara Biotechnology, Kyoto, Japan) was used for cDNA synthesis. SYBR Premix Ex Taq II (Cat# RR820A, Takara Biotechnology) was used for quantitative PCR, which was conducted on a StepOneTM Real-time PCR System (Cat# 4,376,373, Thermo Fisher Scientific). The thermocycling programs: 95 ℃ 10 min, 95 ℃ 30 s then 60 ℃ 1 min 40 cycle. GAPDH was used as the reference gene. Table 1 shows the base sequences of the primers used.

**Table 1:** The primers for RT-PCR

Genes	Forward primer (5′-3′)	Reverse primer (5′-3′)
HBE	GCCTGTGGAGCAAGATGAAT	GCGGGCTTGAGGTTGT
HBG	CTGACTTCCTTGGGAGATGC	TCCCAGGAGCTTGAAGTTCTC
HBB	TACATTTGCTTCTGACACAAC	ACAGATCCCCAAAGGAC
GATA1	CACGACACTGTGGCGGAGAAAT	TTCCAGATGCCTTGCGGTTTCG
EPOR	TTCTGTGCTTCACCGAGCGGTT	CGACACAGCTTCCATGGCTCAT
KLF1	TTGCGGCAAGAGCTACACCAAG	GTAGTGGCGGGTCAGCTCGTC
GYPA	ATATGCAGCCACTCCTAGAGCTC	CTGGTTCAGAGAAATGATGGGCA
BCL2	ATCGCCCTGTGGATGACTGAGT	GCCAGGAGAAATCAAACAGAGGC
TP53	CCTCAGCATCTTATCCGAGTGG	TGGATGGTGGTACAGTCAGAGC
PTEN	TGAGTTCCCTCAGCCGTTACCT	GAGGTTTCCTCTGGTCCTGGTA
ABL1	CCAGGTGTATGAGCTGCTAGAG	GTCAGAGGGATTCCACTGCCAA
MAPK1	ACACCAACCTCTCGTACATCGG	TGGCAGTAGGTCTGGTGCTCAA
CASP3	GGAAGCGAATCAATGGACTCTGG	GCATCGACATCTGTACCAGACC
GAPDH	CATTTCCTGGTATGACAACGA	CTTCCTCTTGTGCTCTTGCT

### Flow-cytometry assay

For intracellular antigens, freshly harvested cells were fixed and permeated using a commercialized kit (Cat# 554,714, BD Bioscience, NJ, USA) and labeled with HbF (Cat# MHFH01 FITC, Thermo Fisher Scientific) and HBB (Cat# sc-21757 PE, Santa Cruz, TX, USA). For the cell surface antigens, cells were directly labeled with CD235a (Cat# 349,114, Biolegend, CA, USA) and CD71 (Cat# 334,104, Biolegend). The positive ratio and median fluorescence intensity (MFI) were assayed by 10^4^ recorded events of flow-cytometry analysis (Attune NxT Acoustic Focusing Cytometer, A24863, Thermo Fisher Scientific).

### Western blot analysis

Proteins were lysed from cell samples by RIPA lysis buffer (Cat# P0013B, Beyotime, Shanghai, China) and quantified by a BCA protein concentration assay kit (Cat# PC0020, Solarbio, Beijing, China). Thirty micrograms of protein was loaded, separated by sodium dodecyl sulfate–polyacrylamide gel electrophoresis (SDS-PAGE), and transferred to polyvinylidene fluoride (PVDF) membranes for antibody blotting. The primary antibodies were ATG7 (Cat# 67,341–1-Ig) and BECN1 (Cat# 11,306–1-AP), purchased from Proteintech (Wuhan, China). GAPDH (Cat# ab59164, Abcam, Cambridge, MA, USA) was used as a control. Primary antibody incubations were performed at a dilution of 1:2000 in PBS for 16 h at 4 ℃. The secondary antibodies were purchased from Yeasen Biotechnology (Shanghai, China) and included peroxidase-conjugated goat anti-rabbit (1:5000 dilution, Cat# 33101ES60) and anti-mouse IgG (1:5000 dilution, Cat# 33201ES60). Secondary antibody incubations were 1 h at room temperature.

### Bioinformatics analysis of mRNA expression

The mRNA expression of K562 cells and HSCs was downloaded from the GEO database GSE48558 series (https://www.ncbi.nlm.nih.gov/geo/query/acc.cgi?acc=GSE48558); GSM1180799, GSM1180802 and GSM1180805 were used for K562 cells, and GSM1180849, GSM1180853 and GSM1180857 were used for CD34-positive HSCs. All samples of GSE48558 were conducted on Affymetrix Human Gene 1.0 ST Array platform, and the online data already have been normalized for further analysis. Gene expression was analyzed using the ‘limma’ package (R package version 3.34.9). The cutoff threshold of DEGs was set as a false discovery rate (FDR) < 0.05 and |log2-fold change (FC)|> 1. Kyoto Encyclopedia of Genes and Genomes (KEGG) signaling pathway [20–22] and GO (Gene Ontology) enrichment analyses were adopted to reveal potential mechanisms in these intersection genes using the ‘clusterProfiler’ package in R. Gene set enrichment analysis (GSEA) was also analyzed using the ‘clusterProfiler’ package. Based on the results of enrichment analysis, we used hypergeometric test to evaluate the degree of enrichment for a specific pathway. The hypergeometric test takes into account factors such as the total number of genes with KEGG/GO annotations in the genome, the total number of genes belonging to that specific pathway in the genome, the total number of differentially expressed genes with KEGG/GO annotations, and the number of differentially expressed genes belonging to that specific pathway. To control errors caused by multiple comparisons, we used the Benjamini–Hochberg method for multiple hypothesis testing correction to calculate the q-value. We defined a pathway as significantly enriched if its q-value was less than 0.05.

### Wright-Giemsa staining

The fresh erythrocytes derived from HSCs were spread directly on the coverslip and blown dry in a fume hood. The staining was performed using a Wright-Giemsa staining kit according to the manufacturer’s instructions. (Cat#, BA-4017, Baso, Zhuhai, China). Cell morphology images were recorded using a Nikon microscope TS 100.

### Statistical analysis

The quantitative results are presented using a histogram according to the means ± SD, while the proportional results are expressed by scatter plots. The comparisons of two groups were statistically analyzed using unpaired Student’s *t* tests. The one-way analysis of variance (ANOVA) was used to analyze the significant of groups. The significant of each group was determined by Tukey test (for unequal variances) and Least Significant Difference test (for equal variances). When *p* values < 0.05, the comparison was considered statistically significant.

## Results

### The DEGs of K562 cells and HSCs were associated with the stem cell and erythroid differentiation pathways

**Fig. 1:**
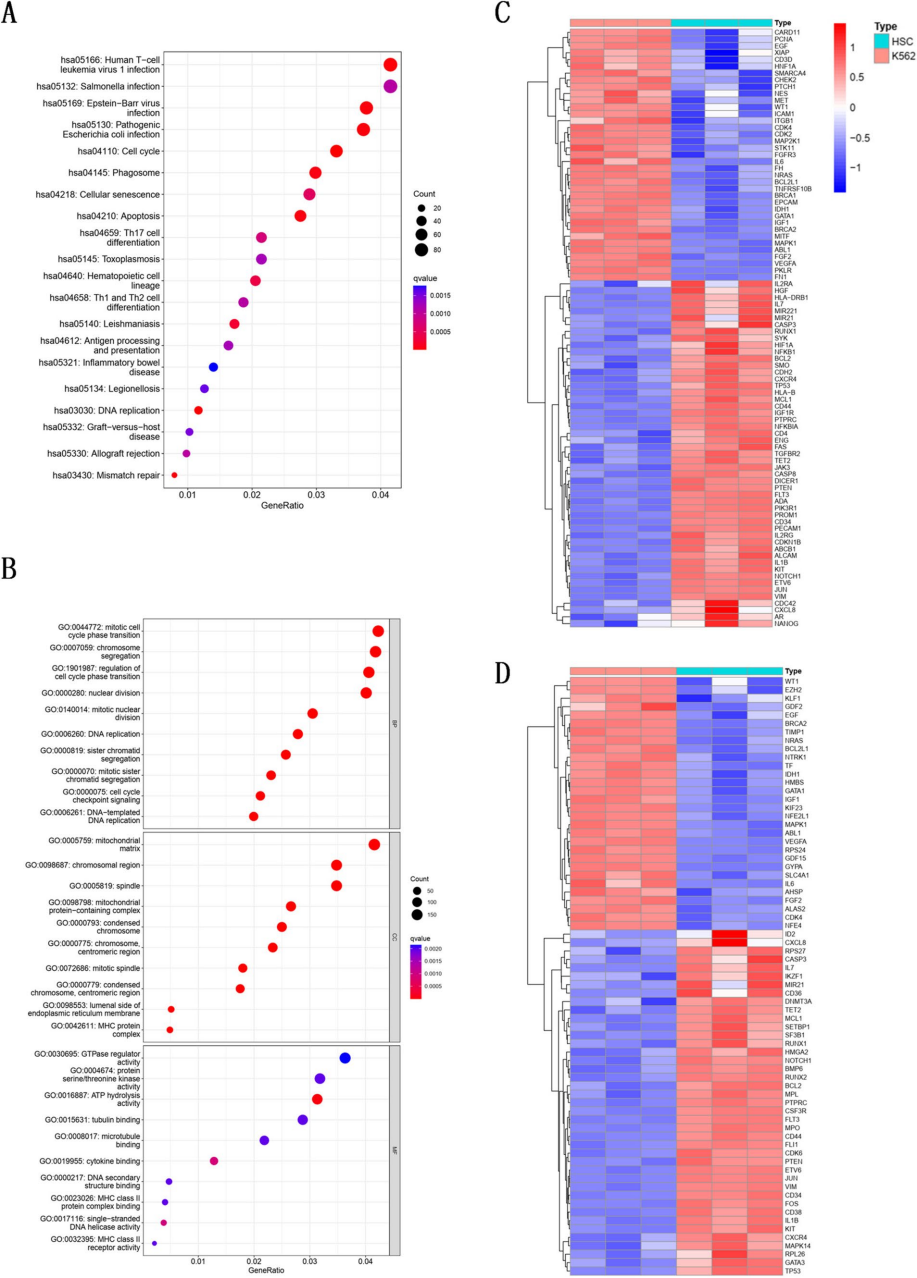
Bioinformatics analysis of the DEGs compared with HSCs and K562 cells using the online data of GSE48558. Differentially expressed genes (DEGs) were used for Kyoto Encyclopedia of Genes and Genomes (KEGG) pathway (**A**) and Gene Ontology (GO) term (**B**) enrichment analysis. The gene counts, q values and GeneRatio of enriched items are presented. The heatmap was used to represent the DEGs of K562 cells and HSCs associated with ‘stem cells’ (*n* = 88) (**C**) and with ‘erythroid differentiation’ (*n* = 71) (**D**)

The top KEGG pathway enrichment analyses of DEGs were associated with infection disease (such as ‘Human T-cell leukemia virus 1 infection’ (hsa05166)), cell differentiation (such as ‘Th17 cell differentiation’ (hsa04659)) and cell death pathway (such as ‘Cellular senescence’ (hsa04218)) (Fig. 1A. In GO term (B) enrichment analysis, some our concerned terms were significant enrich, such as cell cycle for biologic process (BP) (such as ‘mitotic cell cycle phase transition’ (GO:0044772) and ‘regulation of cell cycyle phase transition’ (GO:1,901,987)), energy metabolism agent mitochondrial for cellular component (CC) (such as ‘mitochondrial matrix’ (GO:0005759) and ‘mitochondrial protein-containing complex’ (GO:0098798)) and protein kinase activity for molecular function (MF) (such as ‘GTPase regulator activity’ (GO:0030695) and ‘protein serine/threonine kinase’ (GO:0004674)) (Fig. 1B. The pathway-associated genes were obtained from the web of online (https://www.genecards.org/), and the top 200 score genes associated with ‘stem cell’ and ‘erythroid differentiation’ were used to identify the DEGs (Table S1). Interestingly, an opposite behavior can be observed between K562 and HSCs, as there were 88 DEGs associated with ‘stem cells’ (Fig. 1C) and 77 DEGs associated with ‘erythroid differentiation’ (Fig. 1D).

### Serum starvation promotes erythroid maturation and HBB expression in hemin-induced K562 differentiation

**Fig. 2:**
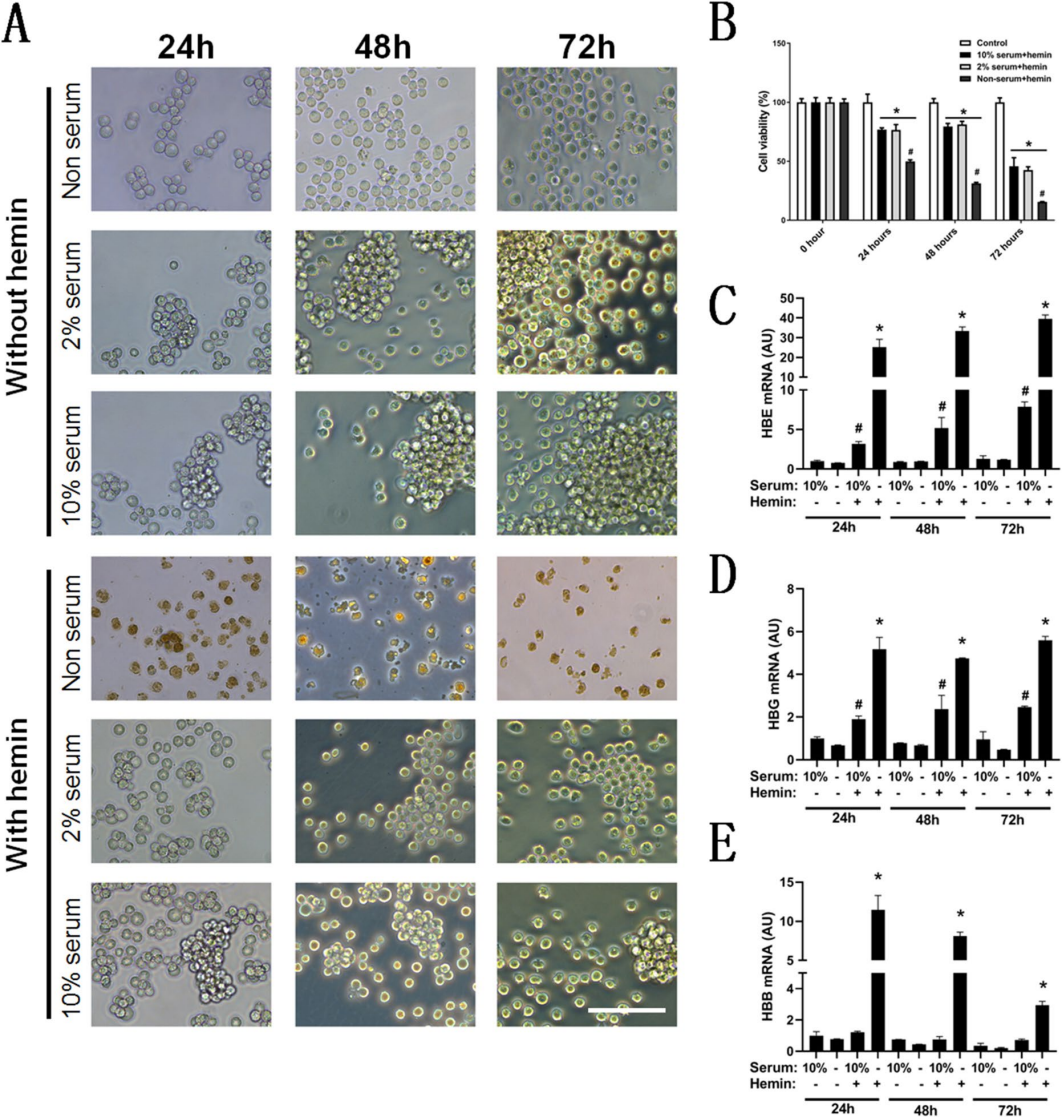
Serum starvation aggravates hemin-induced cell death and promotes erythroid differentiation of K562 cells. **A** Cell morphology observed by bright-field microscopy after hemin-induced erythroid differentiation with different serum ratios in K562 cells; scale bar = 100 μm. **B** The corresponding cell viability assayed by CCK8 (**B**), *n* = 3,* compared with the control group (10% serum culture group) and *p* < 0.05, # compared with the 10% serum + hemin group and *p* < 0.05. RT–PCR was used to analyze the mRNA expression of HBG (**C**), HBE (**D**) and HBB (**E**) with serum-starvation induction, *n* = 3, # compared with the 10% serum culture group and *p* < 0.05, * compared with the 10% serum + hemin group and *p* < 0.05

Erythroid differentiation of K562 cells was induced by 60 μM hemin for 72 h (Fig. 2A). After 72 h of induction, the cell spheres were lost, and the number of cells was decreased with the reduction in serum, regardless of hemin induction (Fig. 2A). Some cells were disintegrated and turned red in the nonserum group with hemin induction (Fig. 2A). Cell viability was assessed by the CCK8 assay, which indicated that cells were significantly reduced in the differentiation groups compared with the control group and in the nonserum differentiation group compared with the 10% serum differentiation group (Fig. 2B). RT-PCR was used to identify the mRNA expression of globins. There were similar results for HBE (Fig. 2C) and HBG (Fig. 2D) mRNA expression, as it was significantly increased in the 10% serum differentiation group compared with the 10% serum culture group and in the nonserum differentiation group compared with the 10% serum differentiation group. Regarding HBB mRNA expression (Fig. 2E), there was no significant change compared with that in the 10% serum culture, nonserum culture and 10% serum differentiation groups, and a significant increase was observed in the nonserum differentiation group compared with the 10% serum differentiation group.

**Fig. 3:**
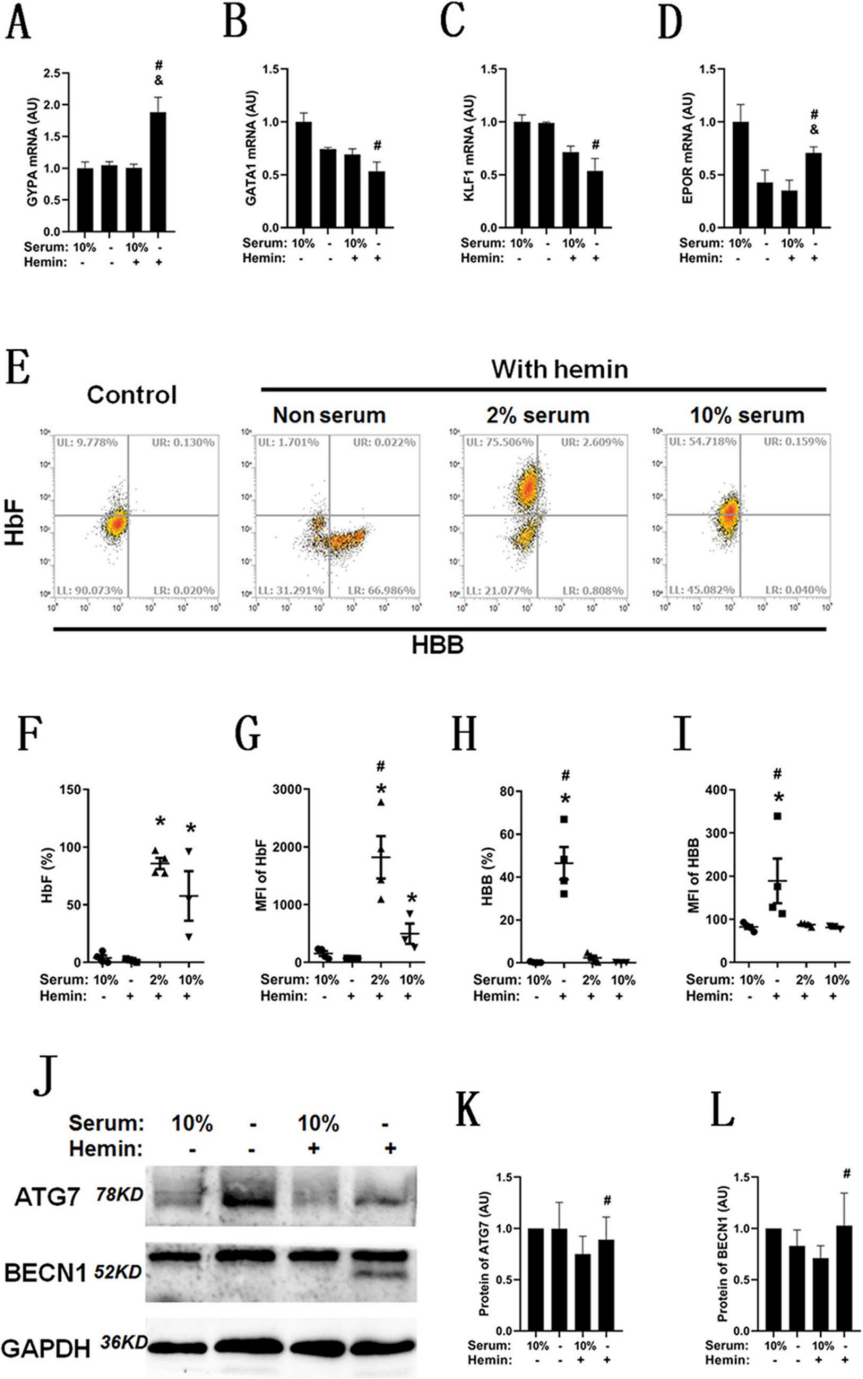
Serum starvation promotes GYPA and EPOR mRNA expression and leads to the globin switch. RT-PCR was used to analyze the mRNA expression of GYPA (**A**), GATA1 (**B**), KLF1 (**C**) and EPOR (**D**) after K562 cells were treated with hemin for 24 h, *n* = 3, # compared with the 10% serum culture group and *p* < 0.05, & compared with the 10% serum + hemin group and *p* < 0.05. Flow cytometry was used to analyze HbF and HBB expression after K562 cells were treated with hemin for 72 h (**E**). Statistical analysis of the HbF-positive ratio (**F**), median fluorescence intensity (MFI) of HbF (**G**), HBB-positive ratio (**H**) and MFI of HBB (**I**) after K562 cells were treated with hemin for 72 h, *n* = 4, * compared with the 10% serum culture group and *p* < 0.05, # compared with the 10% serum + hemin group and *p* < 0.05. Western blot analysis of the protein levels of ATG7 and BECN1 with or without serum after K562 cells were treated with hemin for 24 h (**J**), and the statistical analysis of protein levels was performed according to the gray density of bands, **K** for ATG7 and **L** for BECN1, *n* = 4, # compared with the 10% serum + hemin group and *p* < 0.05

The process of erythroid differentiation is characterized by loss of nucleus, indicating the low abundance of mRNA in the mature red blood cells due to the enucleation process. Therefore, we selected specific time point for different assay, as the mRNA and protein expressions of erythroid differentiation process were assayed for 24 h, and the protein expression of mature red cells were assayed for 72 h. GYPA (also known as CD235a) is a mature marker of erythrocytes, and its mRNA expression was significantly increased in the nonserum differentiation group compared with the 10% serum culture group and the 10% serum differentiation group (Fig. 3A). Those potential associated markers of erythroid differentiation were also assayed and named GATA1 (Fig. 3B), KLF1 (Fig. 3C) and EPOR (Fig. 3D). All of these markers were significantly decreased in the nonserum differentiation group compared with the 10% serum culture group, while EPOR was significantly increased in the nonserum differentiation group compared with the 10% serum differentiation group. Flow cytometry was used to assay the protein expression of HbF and β-globin (Fig. 3E). The positive ratio (Fig. 3F) and MFI (Fig. 3G) of HbF were significantly increased in the serum differentiation group but not in the nonserum group compared with the 10% serum culture group, and the positive ratio (Fig. 3H) and MFI (Fig. 3I) of HBB were significantly increased in the nonserum differentiation group compared with the 10% serum culture and 10% serum differentiation groups. Starvation is generally associated with the activation of autophagy. The protein levels of ATG7 and BECN1 were assayed with or without 24-h serum treatment (Fig. 3J), and there was a significant increase in ATG7 (Fig. 3K) and BECN1 (Fig. 3L) in the nonserum group compared with the 10% serum group during hemin induction.

### Saracatinib promotes erythroid differentiation and induces cell death of K562 cells

To understand the mechanism of serum starvation, three autophagy inducers were used to treat hemin-induced K562 erythroid differentiation with 10% serum, namely, saracatinib, vemurafenib, and rapamycin. Screening of these drugs was conducted after 24 h of treatment with K562 cells.

**Fig. 4:**
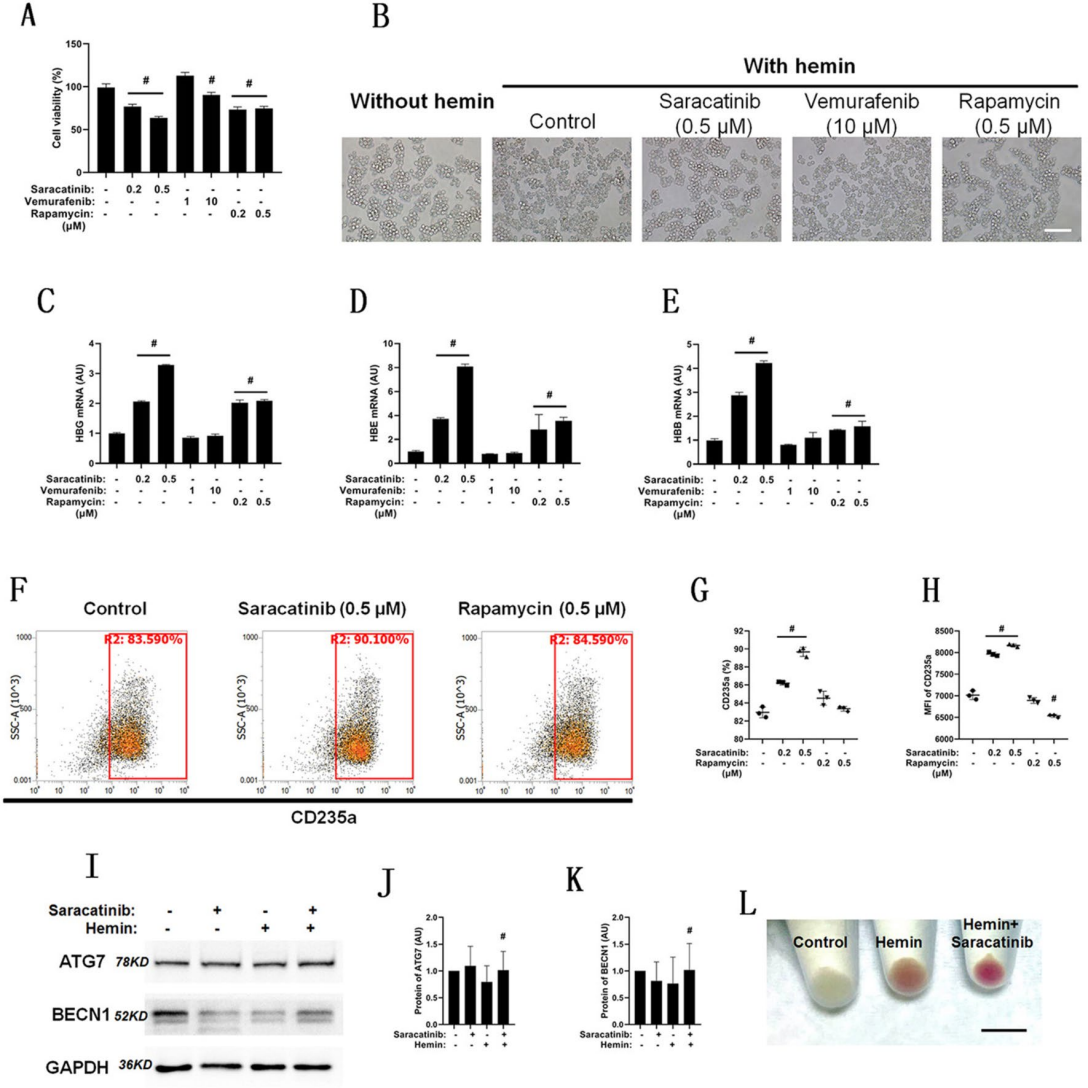
Saracatinib promotes erythroid differentiation of K562 cells. Cell-viability analysis was performed to analyze the cytotoxicity of three autophagy inducers. K562 cells were treated with hemin and one of the inducers in a dose-dependent manner, including saracatinib (0.2 and 0.5 μM), vemurafenib (1 and 10 μM) and rapamycin (0.2 and 0.5 μM), *n* = 3, # compared with the control group and *p* < 0.05 (**A**). (**B**) Cell morphology of K562 cells treated with hemin and each of the drugs for 24 h; scale bar = 100 μm. RT-PCR was used to analyze the mRNA expression of HBG (**C**), HBE (**D**) and HBB (**E**) with the induction of autophagy inducers, *n* = 3, # compared with the control hemin group and* p* < 0.05. CD235a was detected by flow cytometry after K562 cells were treated with hemin and saracatinib or rapamycin for 24 h (**F**), and statistical analysis of the positive ratio (**G**) and MFI (**H**), *n* = 3, # compared with the control hemin group and *p* < 0.05. Western blotting was used to analyze the protein levels of ATG7 and BECN1, while K562 cells were treated with hemin and 0.5 μM saracatinib for 24 h (**I**), and statistical analysis of the protein levels of ATG7 (**J**) and BECN1 (**K**), *n* = 3, # compared with the nonspecific differentiation group and *p* < 0.05. **L** Visual observation of K562 cells induced by hemin with 0.5 μM saracatinib for 72 h; scale bar = 1 cm

The cell viability was analyzed by the addition of each compound in a dose-dependent manner (Fig. 4A), and the cell morphology was recorded after the high-dose treatment (Fig. 4B), as there was no typical change in the comparison of the groups. The mRNA expression of HBG (Fig. 4C), HBE (Fig. 4D) and HBB (Fig. 4E) was assayed. The mRNA expression of each globin was significantly increased by induction with saracatinib and rapamycin but not vemurafenib. Flow cytometry (Fig. 4F) indicated a significant increase in the CD235a-positive ratio (Fig. 4G) and MFI (Fig. 4H) with saracatinib but not rapamycin treatment. The protein levels of ATG7 and BECN1 were assayed after treatment with saracatinib with or without hemin induction (Fig. 4I), and there was a significant increase in ATG7 (Fig. 4. J) and BECN1 (Fig. 4K) in the saracatinib group compared with the nonspecific control group during hemin induction. The cell particles exposed to 0.5 μM saracatinib were smaller and redder than those without saracatinib after 72 h of hemin induction in K562 cells (Fig. 4L). To sum up, we investigated the effects of three autophagy agonists on erythroid differentiation of K562 cells and found that saracatinib promoted erythroid differentiation with a more significant effect than that mediated by the other two autophagy activators. So we chose saracatinib for the following study.

**Fig. 5:**
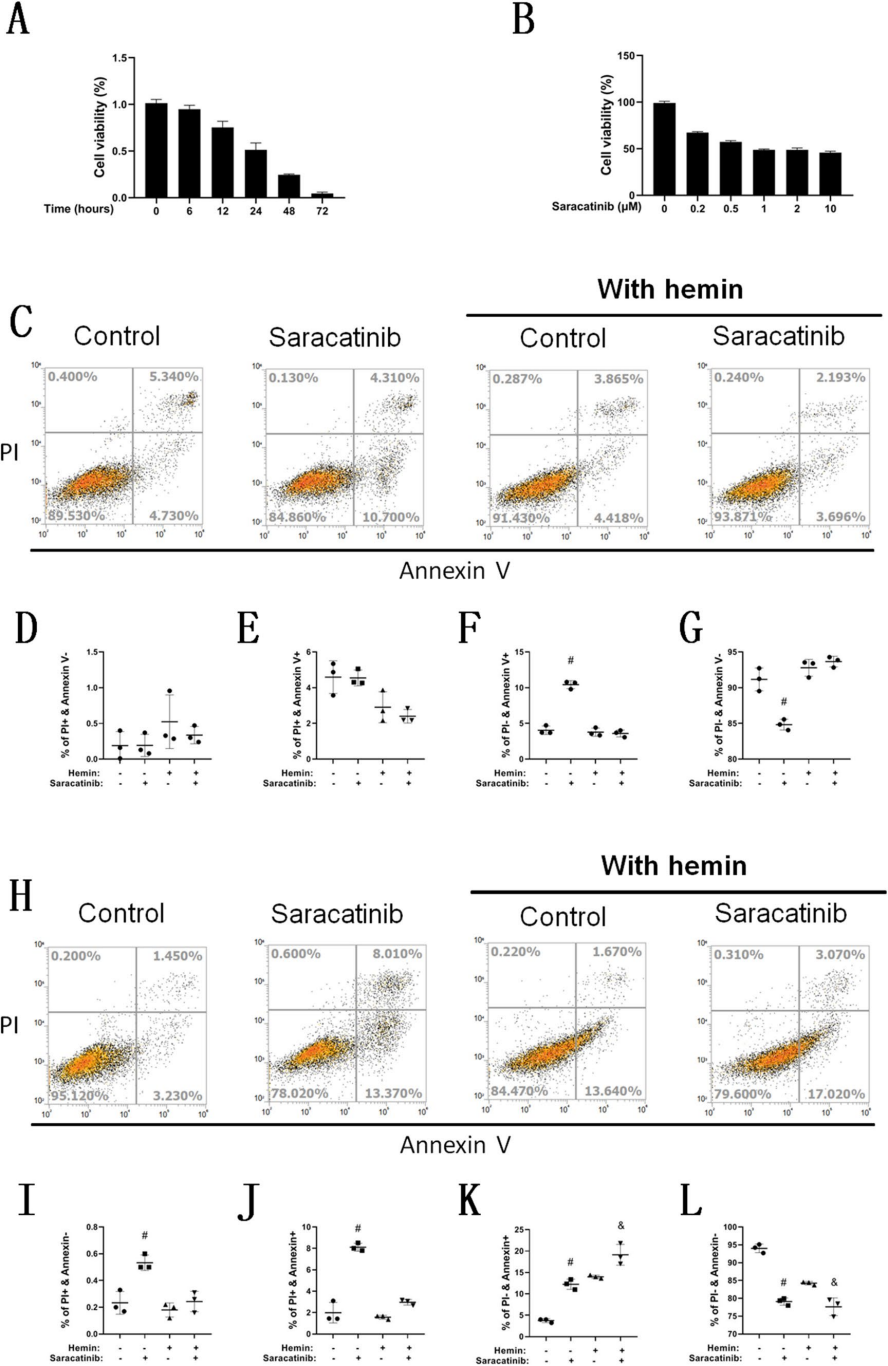
Saracatinib induces K562 cell death. The death of K562 cells induced by saracatinib was analyzed by the CCK8 assay in a time-dependent (**A**) and dose-dependent manner (**B**), *n* = 5. Flow cytometry was used to analyze PI positivity and Annexin V expression, while K562 cells were treated with 0.5 μM saracatinib with or without hemin for 24 h (**C**, **D**, **E**, **F** and **G**) and 48 h (**H**, **I**, **J**, **K** and **L**). Significant differences in statistical analysis are labeled, *n* = 3, # compared with the control culture group and *p* < 0.05 and & compared with the control hemin group and *p* < 0.05

The cytotoxicity of saracatinib to K562 cells was analyzed by the CCK8 assay, and 0.5 μM saracatinib-induced cell death in a time-dependent manner (Fig. 5A). Saracatinib also induced cell death in a dose-dependent manner with 24 h of induction (Fig. 5B). PI and Annexin V double staining were used to analyze the cell death level after saracatinib induction for 24 h (Fig. 5C, D, E, F and G) and 48 h (Fig. 5H, I, J, K and L). At 24 h, there was a significant increase in PI-/Annexin V + cells in the saracatinib group compared with the control culture group. At 48 h, the cell ratio of PI + or Annexin V + was significantly increased in the saracatinib group compared with the control culture group. Under hemin induction, a significant increase in PI -/Annexin V + was found in the saracatinib group compared with the control group.

### Saracatinib did not promote erythroid differentiation of HSCs

**Fig. 6:**
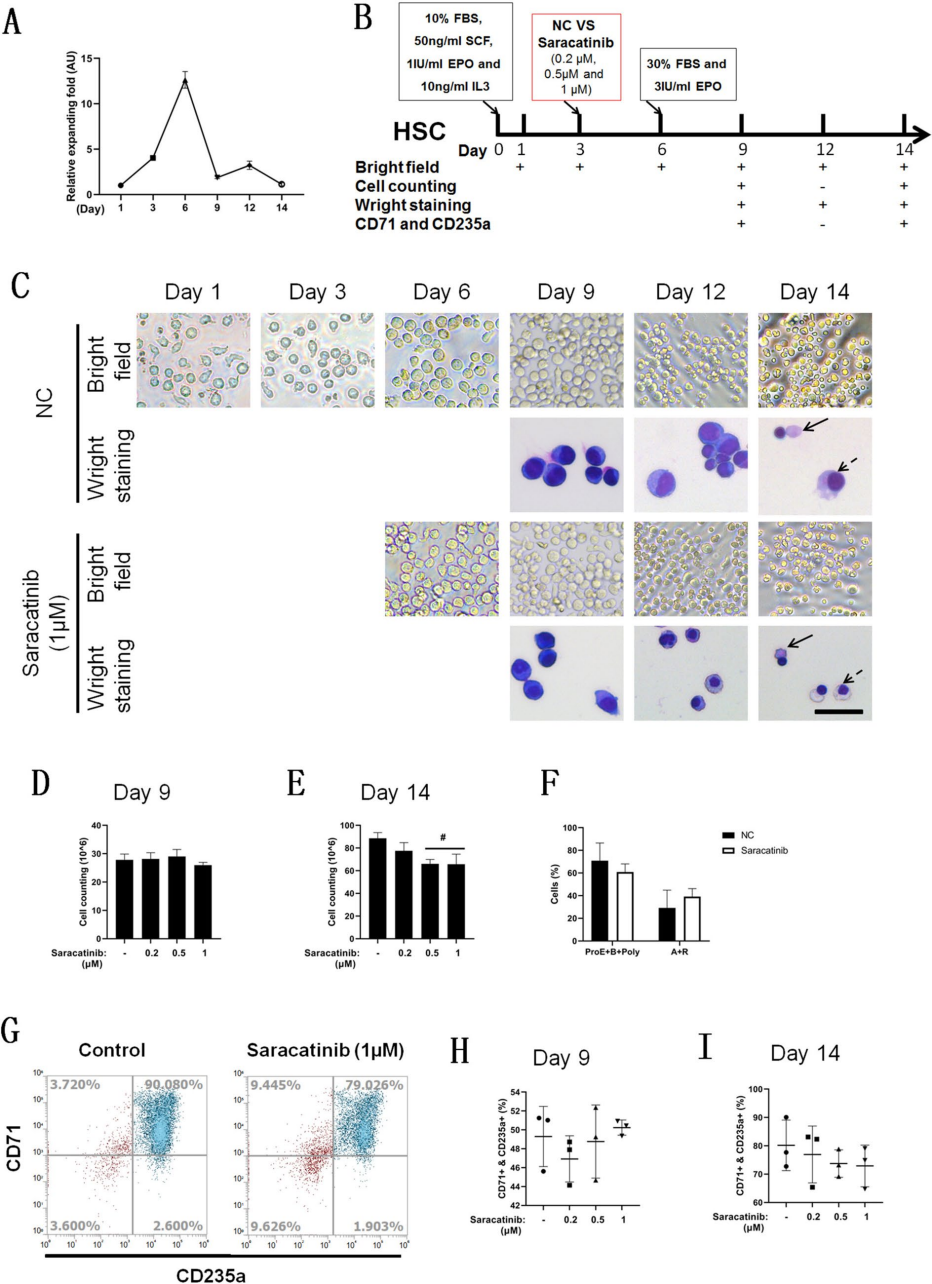
Saracatinib does not promote erythroid differentiation of HSCs. Cell counting was conducted at different times of HSC-committed erythroid differentiation, and a line chart of the relative expanding fold was generated to represent the HSC proliferation rate (**A**). Schematic diagram of the dose-dependent effect of saracatinib on HSC erythroid differentiation (**B**). Bright-field observation and Wright’s staining of HSC erythroid differentiation with or without 1 μM saracatinib (**C**). The figures of lines are in the same magnification, for the first and third lines, scale bar = 100 μm; for the second and fourth lines, scale bar = 20 μm. Regarding cell morphology, the solid arrow represents reticulocytes or acidophilic cells (A + R), and the modest arrow represents polychromatophilic cells or proerythroblasts and basophilic cells (ProE + B + Poly). Statistical analysis of cell counting of different saracatinib groups at Day 9 (**D**) and Day 14 (**E**) of erythroid differentiation, and the percentage of erythrocytes at different stages without or with 1 μM saracatinib at Day 14 (**F**). Flow cytometry was used to assay CD71 and CD235 expression (**G**) (Day 14 for representative) and statistical analysis of ratio of CD71- and CD235-positive cells at Day 9 (**H**) and Day 14 (**I**), *n* = 3, # compared with control differentiation (without saracatinib) and *p* < 0.05

To understand the function of saracatinib in erythroid differentiation of HSCs, the line chart of relative cell fold expansion was generated by counting cells present throughout the entire process of in vitro HSC erythroid differentiation (Fig. 6A). The erythroid differentiation of HSCs was initiated by a rapid cell proliferation period, and over 80% of CD71/CD235a-positive cells were obtained by 14 days of induction. Considering the cytotoxicity of saracatinib, it was added to the 3-day induction as a first-round study, and the assays are listed as a schematic diagram (Fig. 6B). There were no significant differences in cell morphology or cell number with and without saracatinib, and mature erythrocytes were found in the final phase of differentiation, which was identified by Wright staining (Fig. 6C). The cell counts were statistically analyzed on Day 9 (Fig. 6D) and Day 14 (Fig. 6E), and there were significant reductions in HSCs treated with 0.5 μM or 1 μM saracatinib on Day 14, while no significant difference of the mature red cell ratio on Day 14 with saracatinib treatment (Fig. 6F). The expression of CD71 and CD235a was assayed by flow-cytometry analysis (Fig. 6G indicated day 14 with 1 μM Saracatinib treatments for representation), and there was no difference of positive ratio on Day 9 (Fig. 6H) and Day 14 (Fig. 6I).

**Fig. 7:**
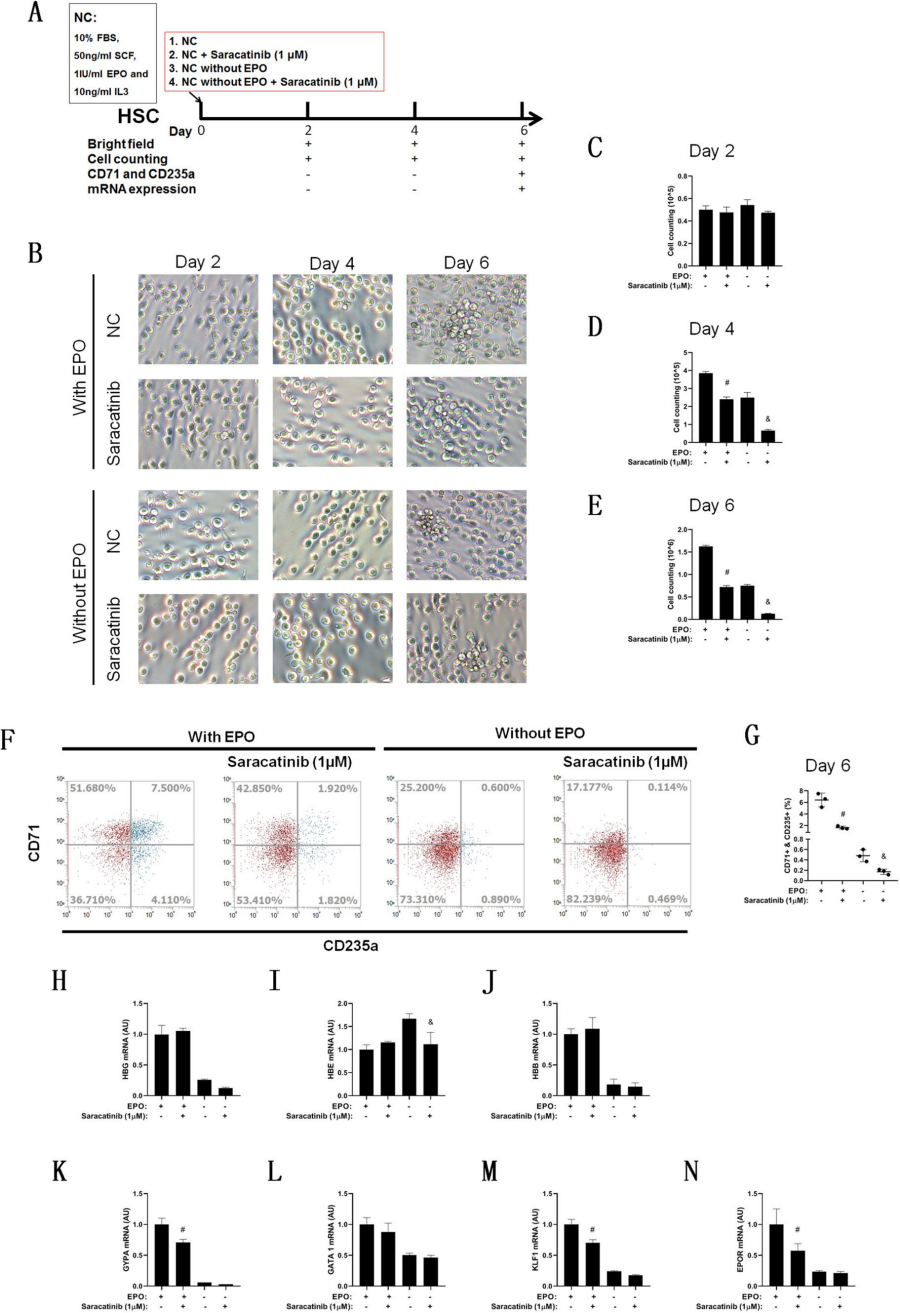
Saracatinib inhibits cell proliferation and erythroid differentiation in the primary stage of HSC erythroid differentiation. **A** Schematic diagram of the study of the effect of saracatinib on the primary stage of HSC erythroid differentiation with or without EPO. Bright-field observation of HSC erythroid differentiation in different groups (**B**), scale bar = 100 μm. Statistical analysis of cell counting was conducted on Day 2 (**C**), Day 4 (**D**) and Day 6 (**E**). The differentiation efficiency was analyzed on Day 6, including the protein expression of CD71 and CD235 (**F** and **G**) and the mRNA expression of HBG (**H**), HBE (**I**), HBB (**J**), GYPA (**K**), GATA1 (**L**), KLF1 (**M**) and EPOR (**N**). *n* = 3, # compared with control differentiation (with EPO) and *p* < 0.05, & compared with differentiation without EPO and* p* < 0.05

As the cytotoxicity of saracatinib to HSCs was not as strong as expected, the second-round study (Fig. 7A) was conducted by adding saracatinib at the initial time of differentiation with and without EPO, which was supplied by serum. There were no typical cell morphology changes in the signaling cells, while cell aggregation was weakened when EPO was withdrawn or saracatinib was added (Fig. 7B). Cell counting results indicated that there were no significant differences on Day 2 (Fig. 7C) and a significant decrease as EPO was withdrawn or saracatinib was added on Day 4 (Fig. 7D) and Day 6 compared with the nonspecific differentiation group (Fig. 7E). Cytoflow (Fig. 7F) indicated that the positive ratio of CD71 and CD235a cells was significantly decreased as EPO was withdrawn or saracatinib was added on Day 6 (Fig. 7G). The level of mRNA expression of globins indicated that saracatinib did not increase their expression with or without EPO (Fig. 7H, I and J). Furthermore, with EPO treatment, saracatinib decreased the mRNA expression of GYPA, KLF1 and EPOR but not GATA1; without EPO, saracatinib did not trigger a significant change (Fig. 7K, L, M and N).

### Saracatinib-induced different mRNA expression changes in erythroid differentiation of K562 cells and HSCs

**Fig. 8:**
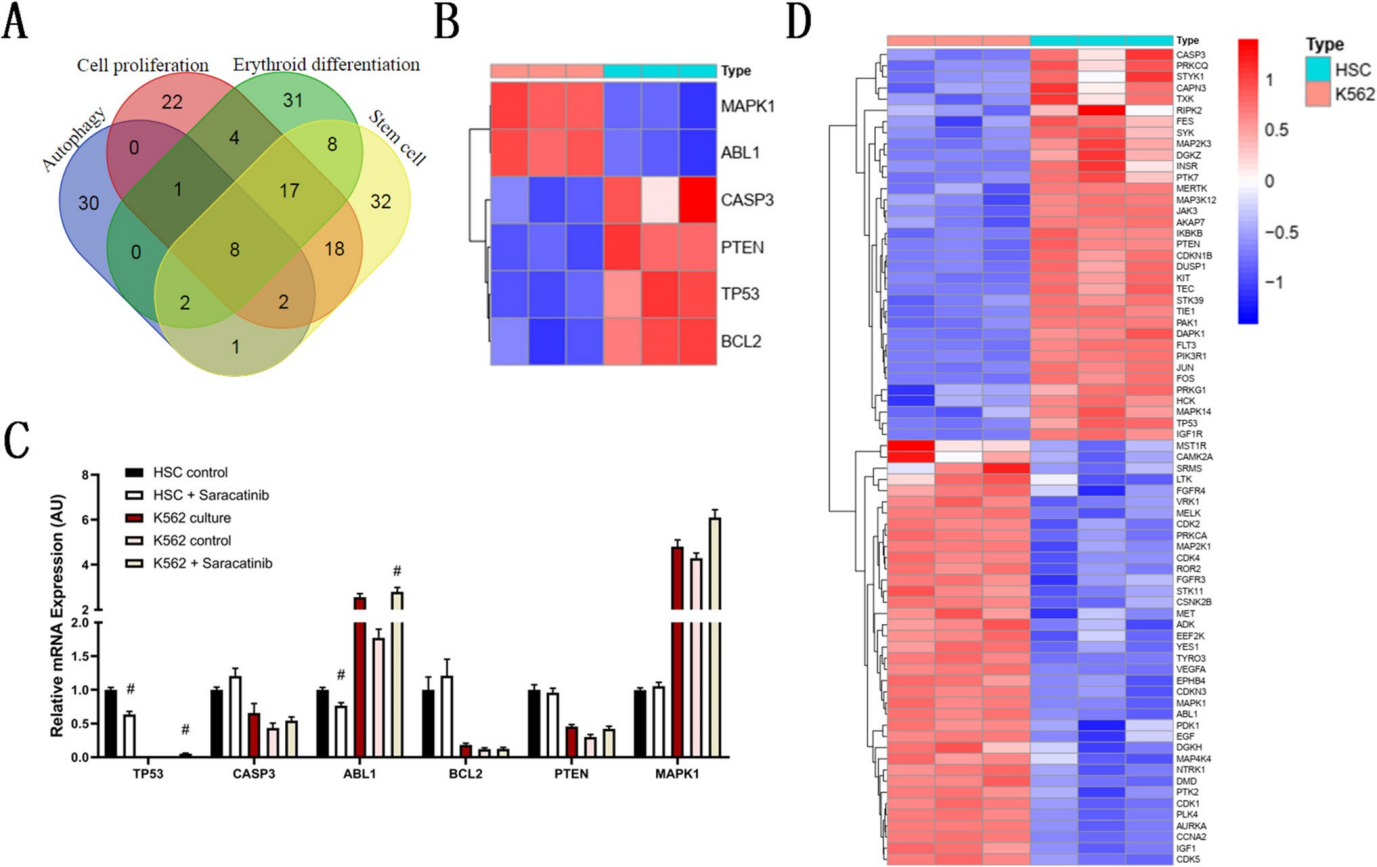
Saracatinib induces differential mRNA expression in K562 cells and HSCs during erythroid differentiation. The DEGs of K562 cells and HSCs associated with ‘autophagy’, ‘cell proliferation’, ‘erythroid differentiation’ and ‘stem cells’ were identified, and eight DEGs were identified as the intersection of the four categories (**A**). The mRNA expression of six DEGs in those eight intersection DEGs was presented in a heatmap, except IL6 and IL1B, which were considered mature markers of immunocytes (**B**). The mRNA expression of those six DEGs was assayed through erythroid differentiation of K562 cells (1 day induced by hemin) and HSCs (6 days induced by cytokine cocktail) with or without saracatinib (**C**), # compared with differentiation without saracatinib and *p* < 0.05. **D** Heatmap of K562 and HSCs DEGs associated with tyrosine-protein kinase (*n* = 73)

To interpret the potential mechanism of saracatinib’s different effects on erythroid differentiation of K562 cells and HSCs, we chose four associated terms to study mRNA expression during erythroid differentiation, namely, ‘autophage’, ‘cell proliferation’, ‘erythroid differentiation’ and ‘stem cell’. The DEGs of each term (Table S2) were used for analysis via a Venn diagram (Fig. 8A). Eight genes were located in the intersection of all terms, namely, BCL2, TP53, PTEN, ABL1, MAPK1, IL6, IL1B and CASP3. As IL6 and IL1B were considered mature markers of immunocytes, the mRNA expression levels of the other 6 DEGs were assessed in a heatmap (Fig. 8B). The relative mRNA expression of those 6 DEGs in K562 cells and HSCs in our study was consistent with the profile in the GEO database. After K562 cells were induced with hemin for 1 day and HSCs were induced with a cytokine cocktail for 6 days, statistical analysis of the mRNA expression of K562 cells and HSCs with or without saracatinib was conducted (Fig. 8C). There were opposite results in K562 and HSC erythroid induction, as the expression of TP53 and ABL1 was significantly decreased with saracatinib in HSC differentiation, while saracatinib induced a significant increase in K562 differentiation. As saracatinib is an inhibitor of tyrosine-protein kinase and ABL1 encodes a nonreceptor tyrosine kinase, tyrosine-protein kinase could be the key pathway leading to the difference in the effect of saracatinib treatment on erythroid differentiation of K562 cells and HSCs. Therefore, there were 73 DEGs associated with ‘tyrosine-protein kinase’ (Fig. 8D) in the mRNA expression profile in K562 cells and HSCs.

## Discussion

The erythroid differentiation of human myeloid leukemia cells is different from that of HSCs. K562 is a human myeloid leukemia cell line that is widely used in the study of erythroid differentiation [4, 5]. The erythroid maturation markers and HBG expression were induced by hemin treatment in K562 cells [23]. This cell model has already been used in drug screening [24] and in the study of the molecular mechanisms of cell development [25], as K562 cells share a similar pathway of erythroid cell development with HSCs. However, the difference between normal and malignant hematopoietic cells is due to the mRNA expression profile [26]. Actually, there were kinds of differences between the erythroid differentiation of K562 and HSCs such as the induction condition, the related pathway and the terminal cells. It is difficult to perform all the experiments parallelly in both cell systems., We thus documented the difference of erythropoiesis between K562 cells and HSCs with the treatment of saracatinib. The cytokine cocktail-induced HSC erythroid differentiation in vitro could minimize erythropoiesis in vivo. The erythroid differentiation protocol of HSCs used in our study [27] was divided into two stages, one for early erythroid progenitor proliferation and the other for erythroid maturation. With the induction of this protocol, HSCs can significantly proliferate, gain mature erythrocyte morphology and molecular markers, and form colony-forming units (CFUs) in specific media. K562 cells were derived from the pleural effusion of a female chronic myeloid leukemia (CML) patient, which is one of the most prolific malignant hematopoietic cell lines used. K562 cells can be differentiated along the erythroid, megakaryocytic or monocytic/macrophage cell axes by different inducers, and hemin is specific to erythroid differentiation [28]. With the induction of hemin, K562 cells express mature erythrocyte molecular markers and globins, similar to HSCs; however, some significant differences exist, such as in DNA repair markers [26] and tyrosine kinase [29]. In this study, we demonstrated the differentiation of K562 cells and HSCs through an autophagy inducer (serum starvation or saracatinib), which prompted hemin-induced K562 erythroid differentiation; however, erythropoiesis of HSCs was suppressed by saracatinib as the initial proliferation was inhibited. The potential mechanism was the opposite behavior of DEGs in K562 cells and HSCs. We assayed the mRNA expression of relating genes. Although saracatinib has no effect on some mRNA expression, there were opposite regulations of TP53 and ABL1 during treatment of saracatinib in erythroid differentiation of K562 cells and HSCs.

The erythroid differentiation of K562 cells was governed by autophagy. Autophagy is a cyto-biological process of the clearance of unnecessary organelles, including ribosomes and mitochondria, to provide the necessary energy and matter to reuse [30]. Classical autophagy inducers, such as serum starvation and rapamycin treatment, increase the mRNA expression of α-globin and γ-globin and the ratio of hemoglobin-positive cells [31]. The erythroid differentiation of K562 cells was characteristic of the induction of α-, γ-, ε- and ζ-globin [28] but not β-globin. During erythropoiesis, GATA1 directly activates the transcription of autophagy-related genes [32], but few studies have addressed the detailed mechanism of KLF1 [33] or EPOR [34] activity or activation by autophagy. EPOR could activate autophagy in some specific physiologic and pathologic conditions [35–37] but not in the erythropoiesis process. In the current study, all β-globin cluster mRNAs were triggered, including HBB, HBG and HBE, by serum starvation or saracatinib. The detailed study of serum starvation indicated that the activation of K562 β-globin peptide expression is different from the normal physiologic HPFH; it facilitates the transition of globin from expressing γ-globin to expressing β-globin, while the opposite occurs in HPFH. Meanwhile, EPOR mRNA was selected but not GATA1 or KLF1 mRNA. In other published literature, tyrosine phosphorylation of EPOR (EPOR-phosphotyrosine-343-Stat5 signaling axis) was essential for erythropoiesis in induced anemia [38]. We found that the tyrosine kinase inhibitor [39] saracatinib, as an autophagy inducer, shared a consistent effect with serum starvation and was a better inducer of prompt hemin-induced K562 erythroid differentiation than rapamycin and vemurafenib. K562 is a typical malignant cell line of myeloid leukemia, which committed erythroid differentiation without the presence of EPO. Some other cell lines could mimic HSCs erythroid differentiation better as EPO is participating, such as UT7 or HUDEP. Those cell models could use for monitoring autophagy core proteins after saracatinib treatment and further functional studies are needed to define the role of saracatinib interplay between cell death and red blood cell differentiation. In general, it is concluded that autophagy was triggered by EPO signals in the erythroid differentiation while the different differentiation committed during K562 cells and HSCs. In our study, saracatinib promoted erythroid differentiation in K562 cells, but inhibited it in the early stage of erythroid differentiation in HSCs, and had no effect in the late stage. Consequently, autophagy activators or inhibitors were shown different cellular effect in the erythropoiesis of K562 cells and HSCs.

Tyrosine is one of the basic amino acids that form proteins, and the phosphorylation of tyrosine residues on proteins located on the cell membrane and cytoplasm is an important signal transduction activity. Saracatinib (also known as AZD0530) is a dual tyrosine kinase inhibitor that includes Src and Bcr-Abl [40]. Src family kinases (SFKs) include nine members, encoded by their respective DNA sequences, that regulate the cell events involved in cell proliferation, differentiation and death [41]. Bcr-Abl is an etiological agent of CML and is encoded by a fused DNA sequence of Abl from chromosome 9 and Bcr from chromosome 22, which is formed by chromosome reciprocal translocation and is known as the philadelphia chromosome [42]. The anticancer function of saracatinib was due to the induction of autophagy in solid tumors, such as prostate [43], ovarian [44] and lung [45] cancer; furthermore, saracatinib was also tested in clinical trials for ovarian cancer [46] or small-cell lung cancer [47]. In leukemia cells, saracatinib targets both Src and Bcr-Abl kinase activity and inhibits cell proliferation [48]. We found that the mRNA of ABL1 were reduction of HSCs and induction of K562 cells by saracatinib during erythroid differentiation, and our results indicate that saracatinib prompts hemin-induced K562 erythroid differentiation, accompanied by activation of autophagy and increased cell death, which would be a new anti-leukemia strategy using saracatinib.

In addition to its anticancer efficacy, saracatinib has been used in some other disease treatments. Regarding neurologic diseases, an animal study indicated that saracatinib could alleviate pathologic changes in epilepsy [49], Alzheimer’s disease (AD) [50] and Parkinson’s disease (PD) [51]. However, saracatinib has also been used as an antiviral agent [52] and to treat cardiac dysfunction [53]. Given the wide clinical usage of saracatinib, it is necessary to understand the drug effect mechanism in physiologic and pathologic conditions. In published clinical trials, two independent research institutions covered the side effects of anemia after oral usage of saracatinib [54, 55]. Our results indicated the potential cellular mechanisms of saracatinib-induced anemia, in which HSC proliferation was blocked and HSC erythroid differentiation disorder occurred. The erythroid differentiation of human CD34 + HSCs is a complex BP with precise control of molecular markers, CD105, GPA, α4-integrin and Band3 could be used to distinguish between distinct stages of erythroid differentiation [56], meanwhile, cell morphology change and enucleation [57] were occurred. The role of saracatinib in those processes needs to determine by further study.

Cell transplantation of healthy HSCs was the first choice for the treatment of genetic hemoglobinopathies. Some approaches to genetic modification have already been used in the clinic, such as the repair of pathogenic genes [58], lentiviral transfer of functional HBB genes [59] and active expression of HbF via gene editing [60]. The expression of HBB mRNA is controlled by five cis-acting regulatory elements, which are DNase I hypersensitivity fragments named locus control regions (LCRs). The LCR proximity to HBB promoters by three-dimensional chromosomal interaction, maintains the active chromatin signature and recruitment of key erythroid transcription factors, inducing the transcription and translation of HBB [61]. In addition, activation of adult HbF expression was considered a curative approach, but the understanding of erythroblast F cells and non-F cells (A cells) is limited, as there were no significant differences in F cells and A cells in the transcriptional and proteomic profiling study [62]. In the current study, we demonstrated that autophagy induces a switch of HbF to β-globin in K562 cells. This cell model could be used to study the activation of HBB mRNA transcription and protein translation to achieve a new strategy to cure anemia.

## Conclusion

In summary, the findings described here demonstrate the difference in mRNA expression and the TKI-saracatinib effect on erythroid differentiation between K562 cells and HSCs. Additional studies are needed to determine whether the prompt leukemia cell differentiation effect of saracatinib could be used in anticancer treatment by decreasing the degree of malignancy of the tumor and to further define whether a feasible treatment protocol can be developed to avoid saracatinib’s side effects, such as induced HSC erythroid differentiation disorder.

### Supplementary Information

Below is the link to the electronic supplementary material.

Supplementary file1 (PDF 1209 KB)

Supplementary file2 (XLSX 29 KB)

Supplementary file3 (XLSX 10 KB)

## Data Availability

All data generated or analyzed during this study are included in this published article (and its supplementary information files).
