# Supplementary material for: Saracatinib prompts hemin-induced K562 erythroid differentiation but suppresses erythropoiesis of hematopoietic stem cells
Source: Hum Cell. 2024 Feb 22;37(3):648–65. doi: 10.1007/s13577-024-01034-5 (PMC11016514; doi:10.1007/s13577-024-01034-5)

Figure 3. J

S+: 10% Serum
S-： 0% serum
H+: with Hemin
H-： without hemin

GAPDG

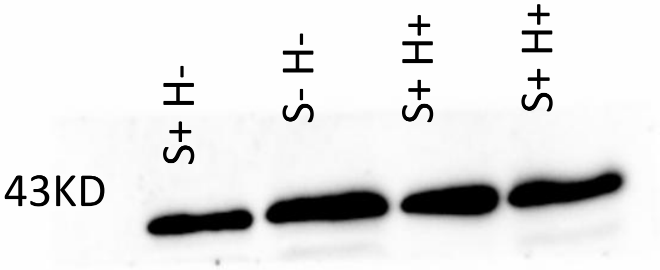

N1

Figure 3. J

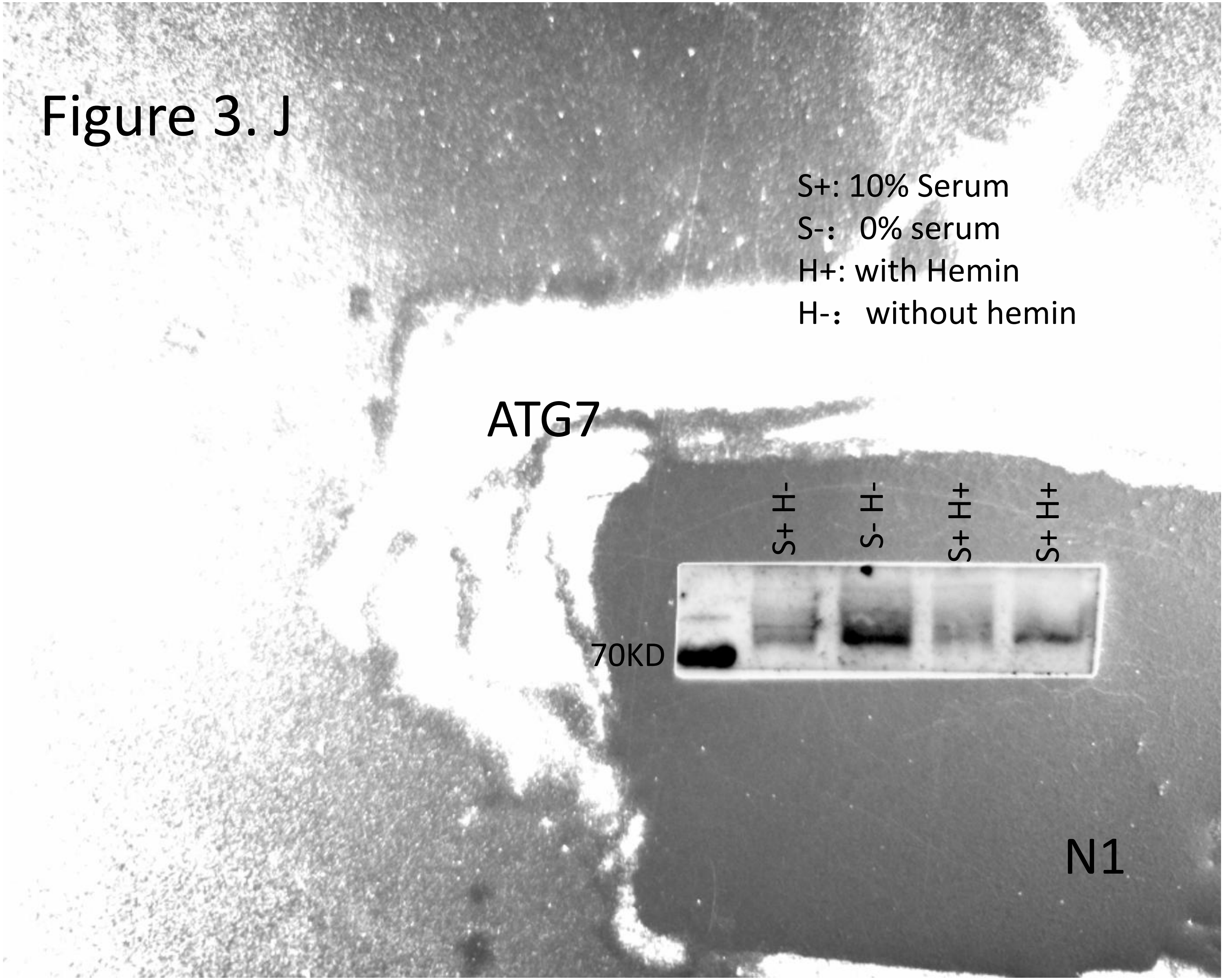
S+: 10% Serum
S-： 0% serum
H+: with Hemin
H-： without hemin
ATG7
S+ H-
S- H-
S+ H+
S+ H+
70KD
N1

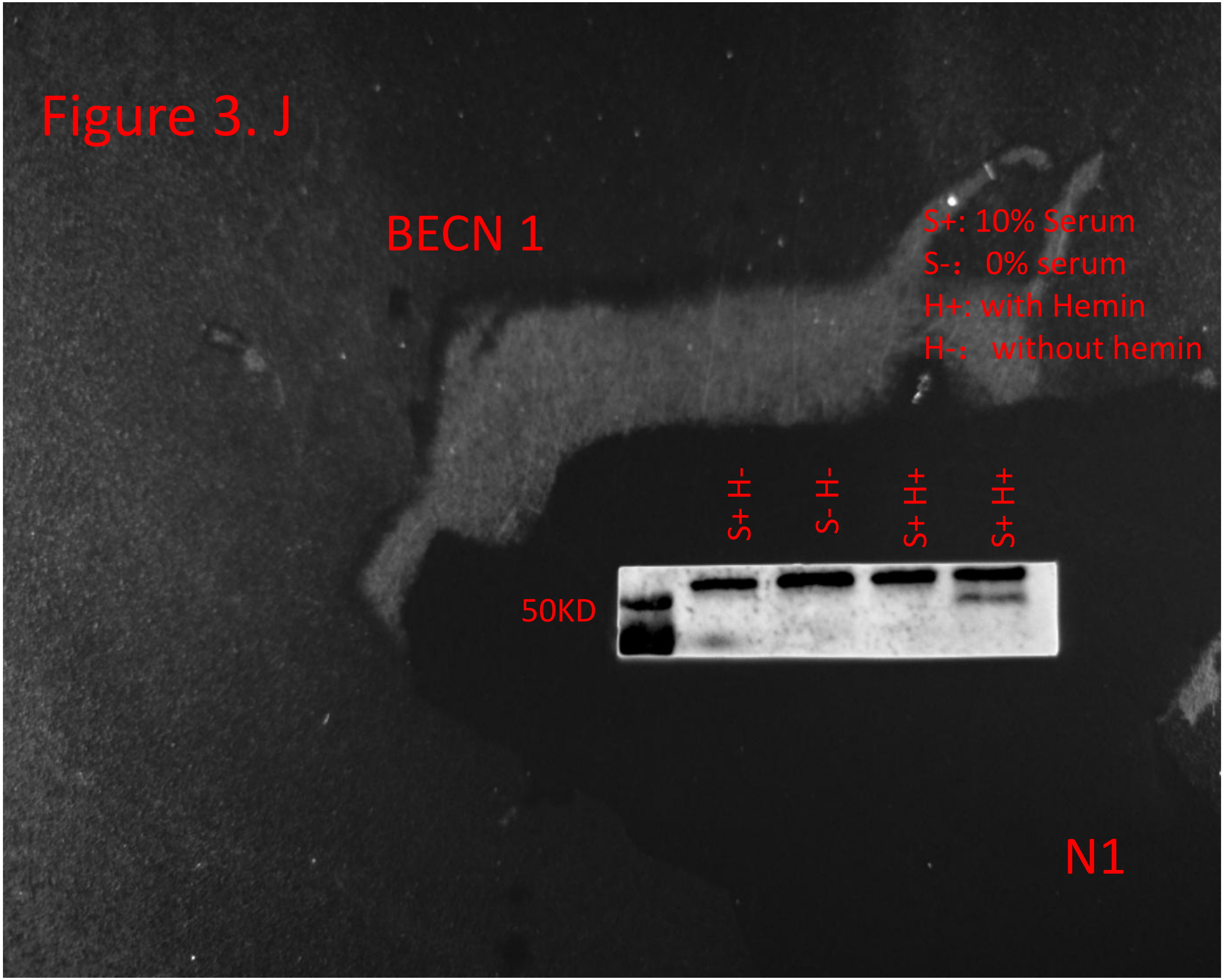
Figure 3. J
BECN 1
S+: 10% Serum
S-: 0% serum
H+: with Hemin
H-: without hemin
S+ H-
S- H-
S+ H+
S+ H+
50KD
N1

Figure 3. J

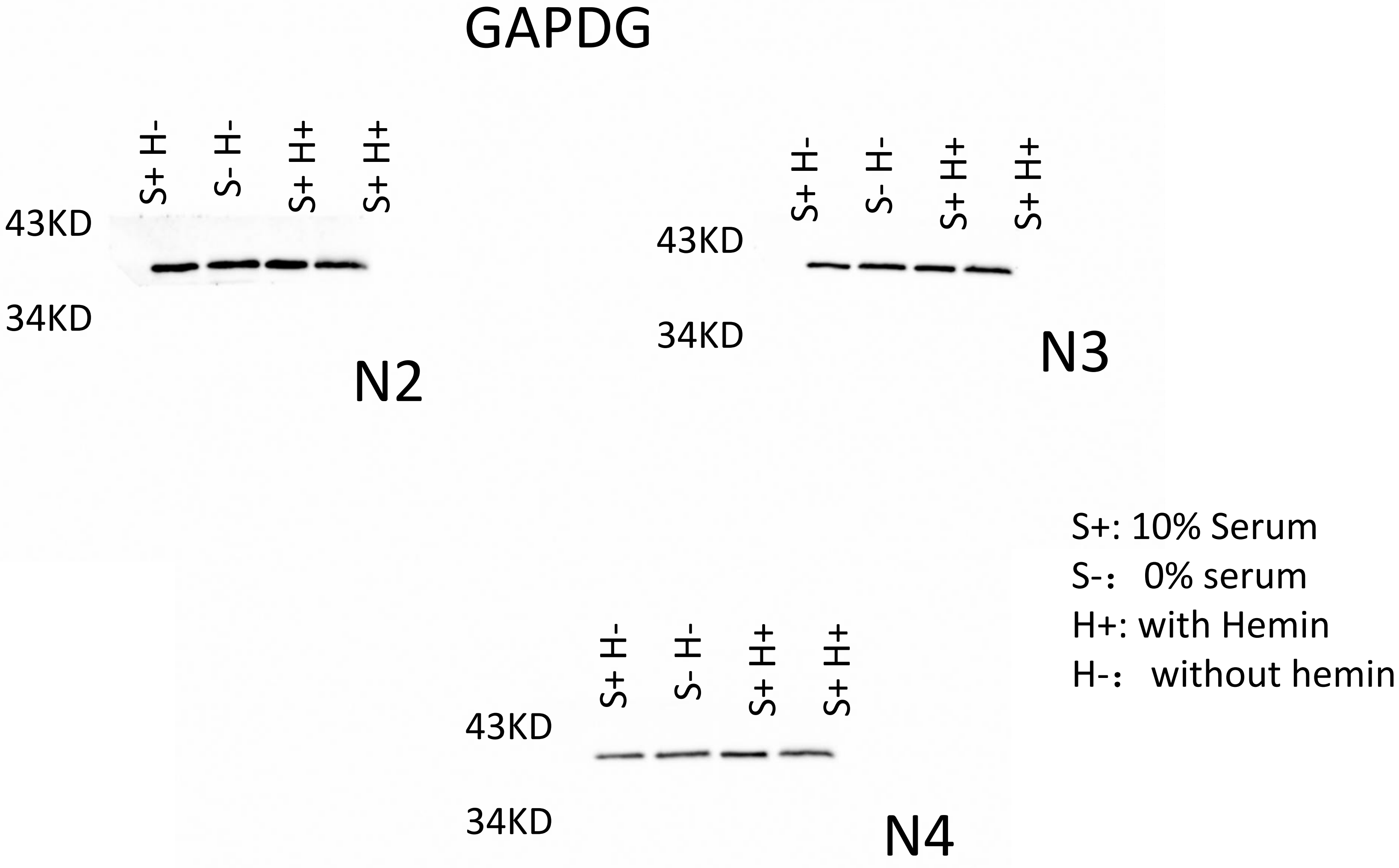
GAPDG
S+ H-
S- H-
S+ H+
S+ H+
43KD
34KD
N2
S+ H-
S- H-
S+ H+
S+ H+
43KD
34KD
N3
S+ H-
S- H-
S+ H+
S+ H+
43KD
34KD
N4
S+: 10% Serum
S-： 0% serum
H+: with Hemin
H-： without hemin

Figure 3. J

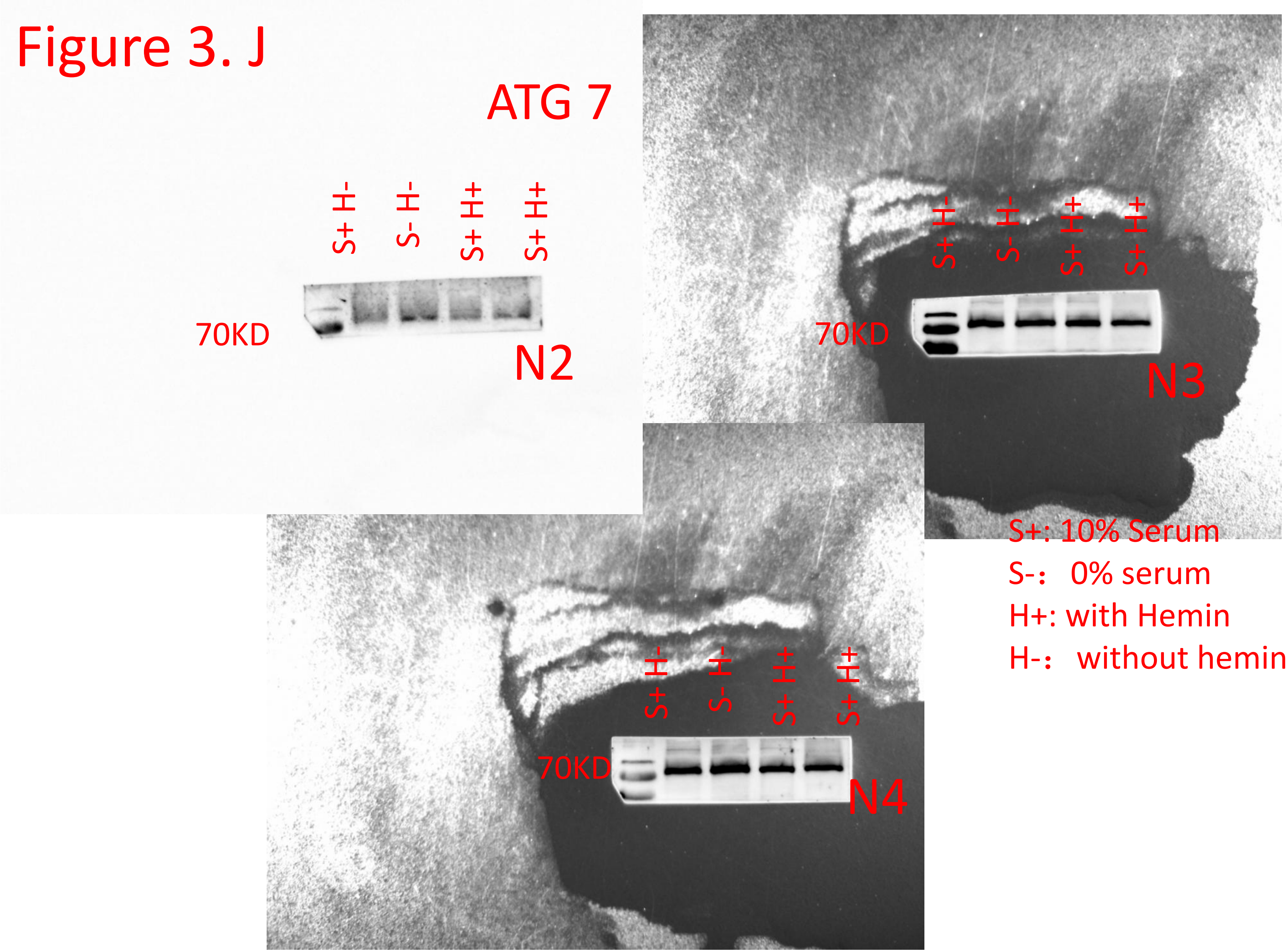

S+: 10% Serum
S-： 0% serum
H+: with Hemin
H-： without hemin

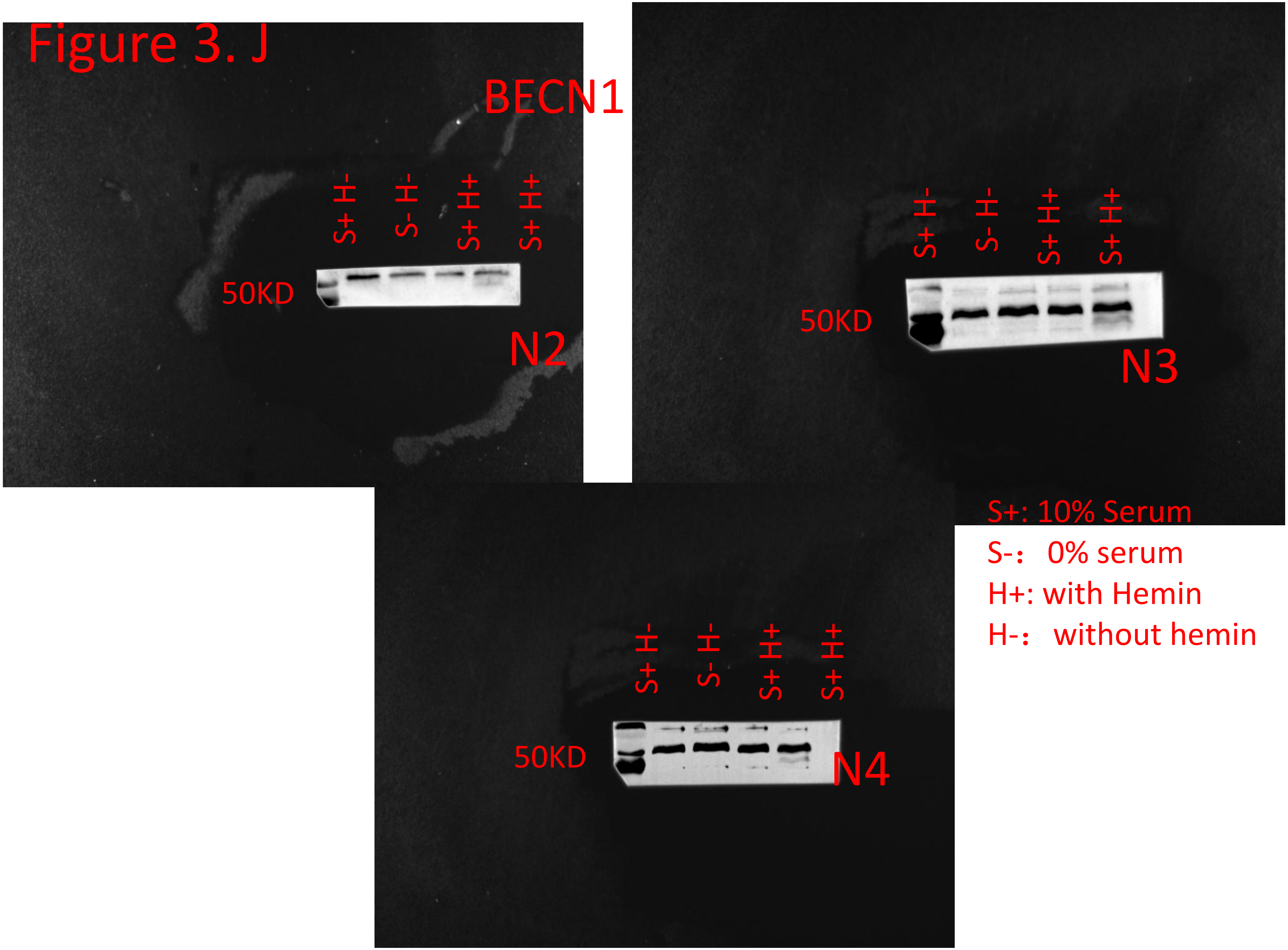

Figure 3. J
BECN1
S+ H-
S- H-
S+ H+
S+ H+
50KD
N2
S+ H-
S- H-
S+ H+
S+ H+
50KD
N3
S+ H-
S- H-
S+ H+
S+ H+
50KD
N4
S+: 10% Serum
S-： 0% serum
H+: with Hemin
H-： without hemin

Figure 4. H

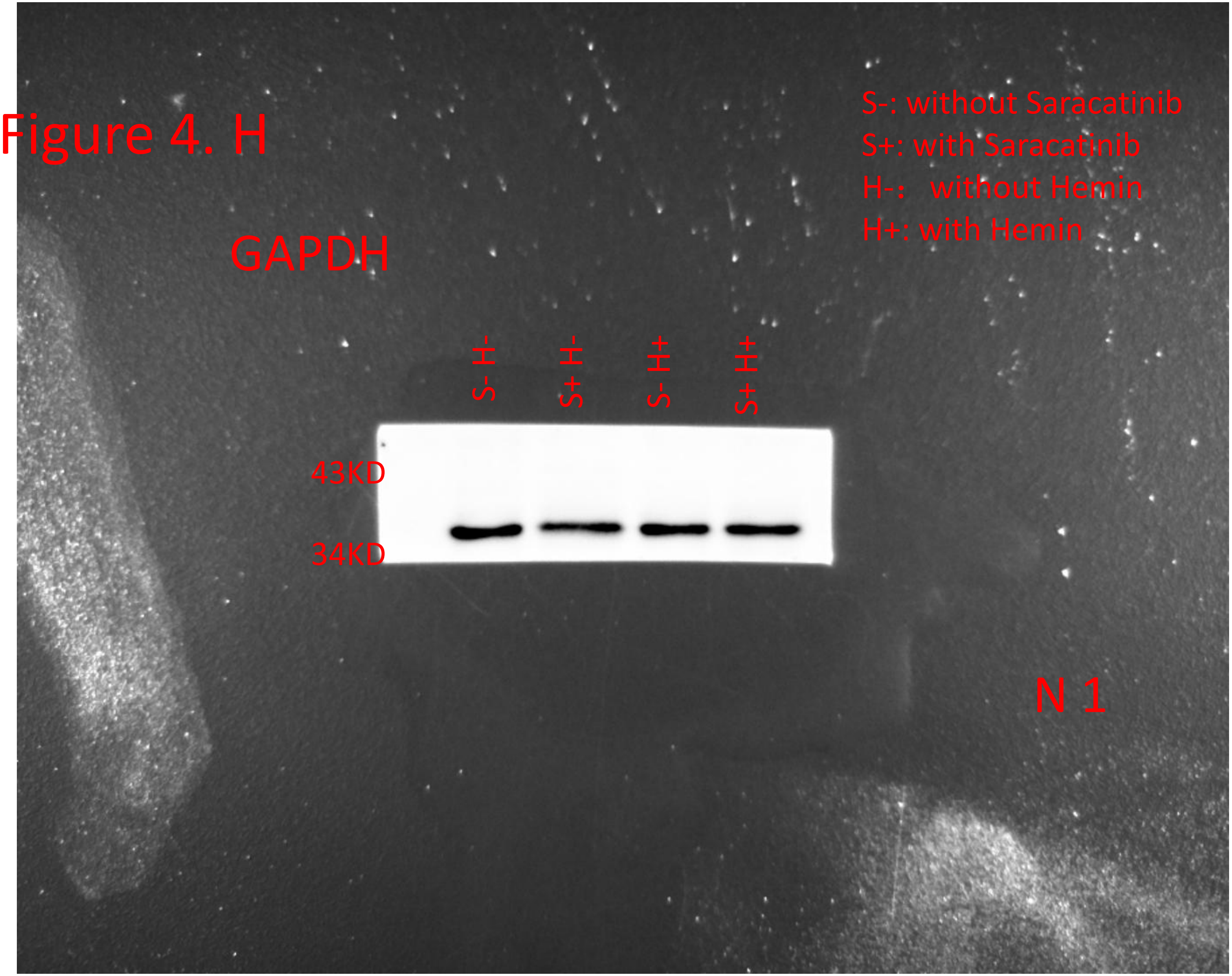

GAPDH
S-: without Saracatinib
S+: with Saracatinib
H-: without Hemin
H+: with Hemin
S- H-
S+ H-
S- H+
S+ H+
43KD
34KD
N 1

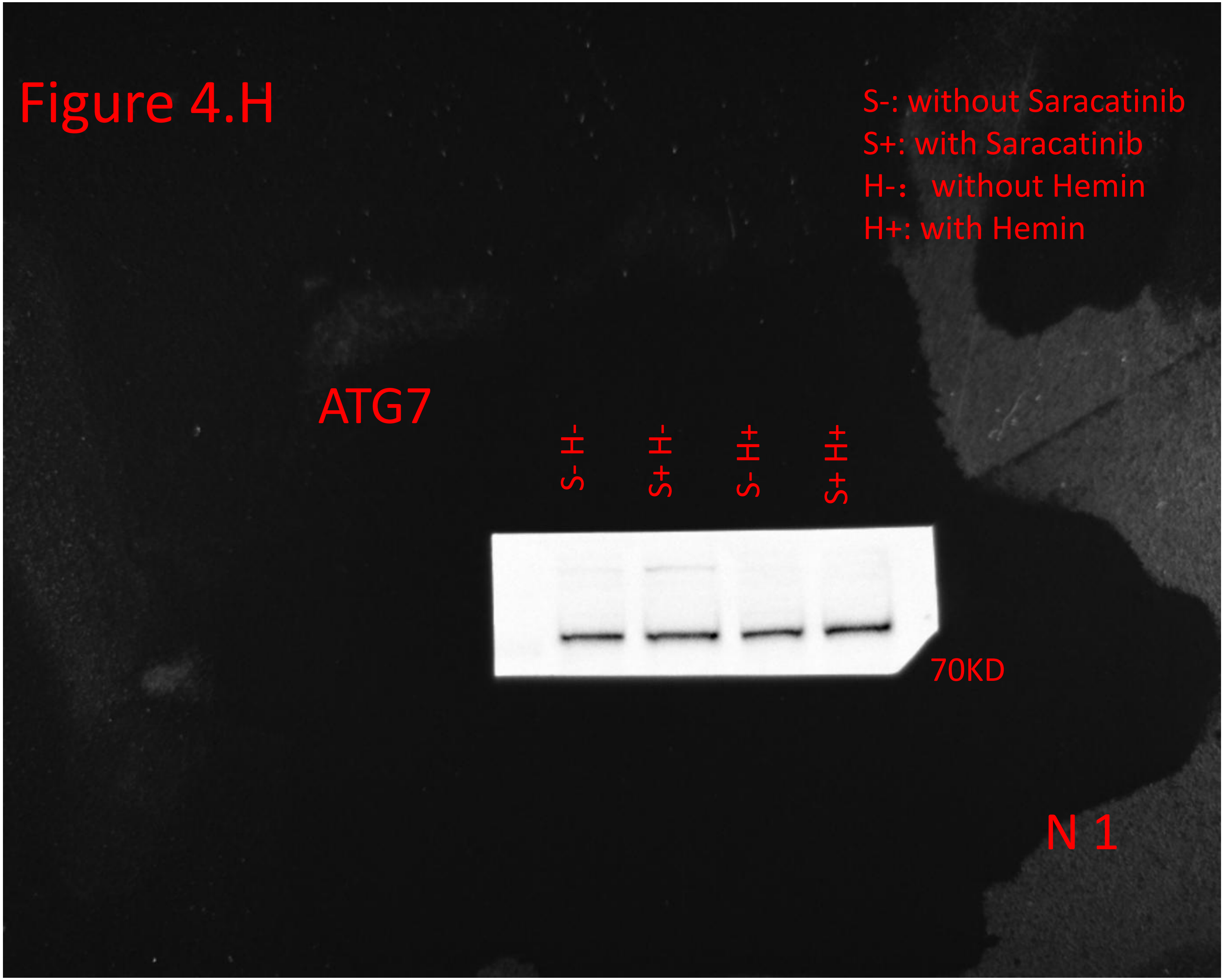
Figure 4.H
S-: without Saracatinib
S+: with Saracatinib
H-: without Hemin
H+: with Hemin
ATG7
S- H-
S+ H-
S- H+
S+ H+
70KD
N 1

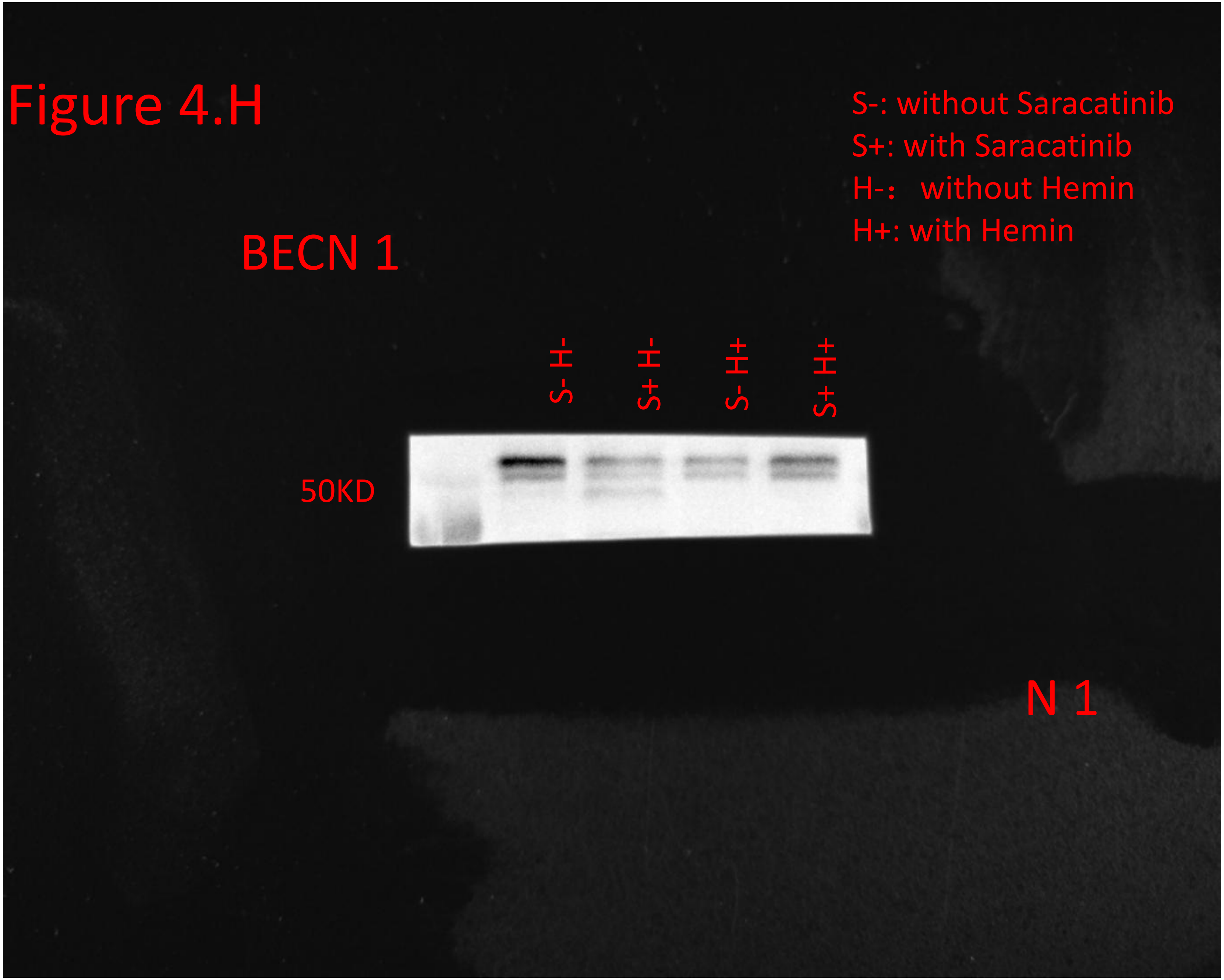
Figure 4.H
S-: without Saracatinib
S+: with Saracatinib
H-: without Hemin
H+: with Hemin
BECN 1
S- H-
S+ H-
S- H+
S+ H+
50KD
N 1

Figure 4. H

GAPDH

S-: without Saracatinib
S+: with Saracatinib
H-： without Hemin
H+: with Hemin

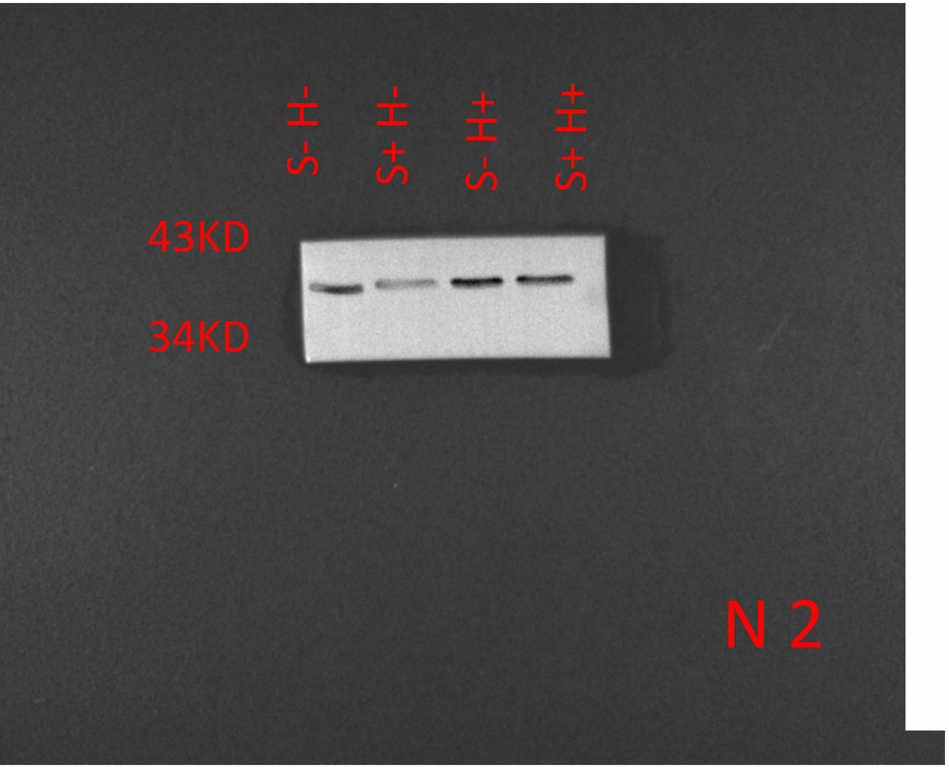

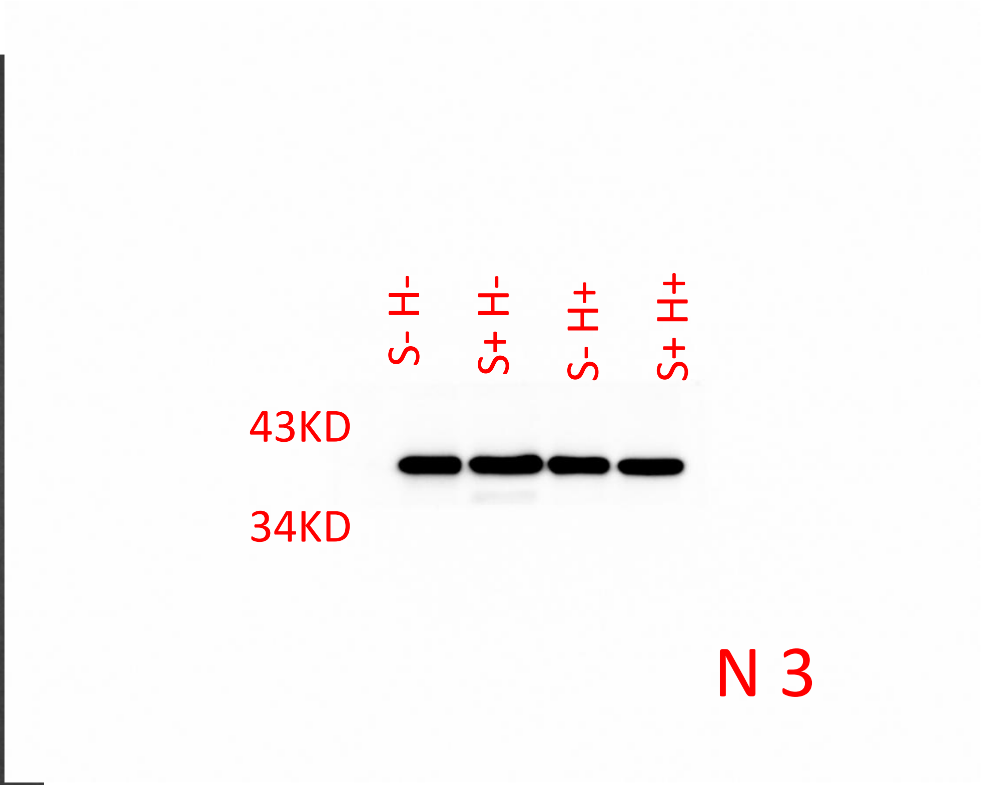

Figure 4. H

ATG7

S+: 10% Serum
S-： 0% serum
H+: with Hemin
H-： without hemin

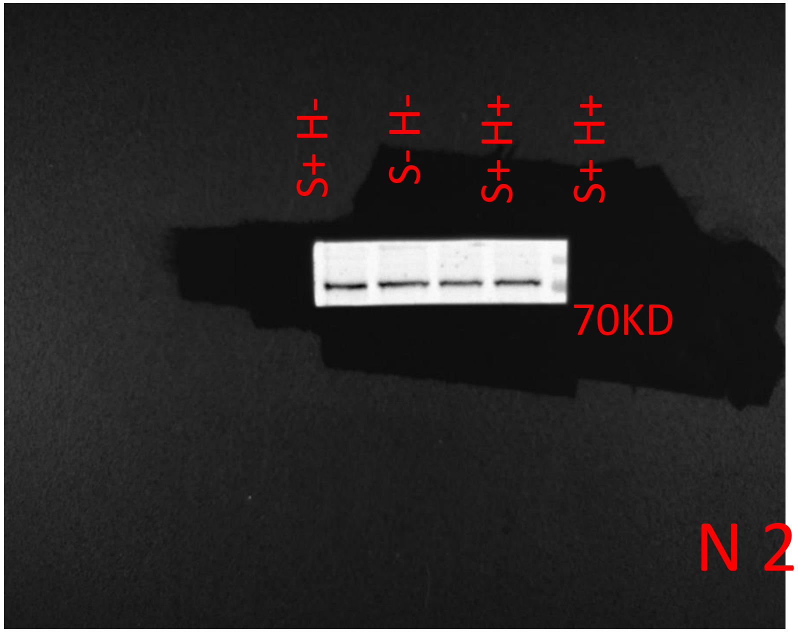

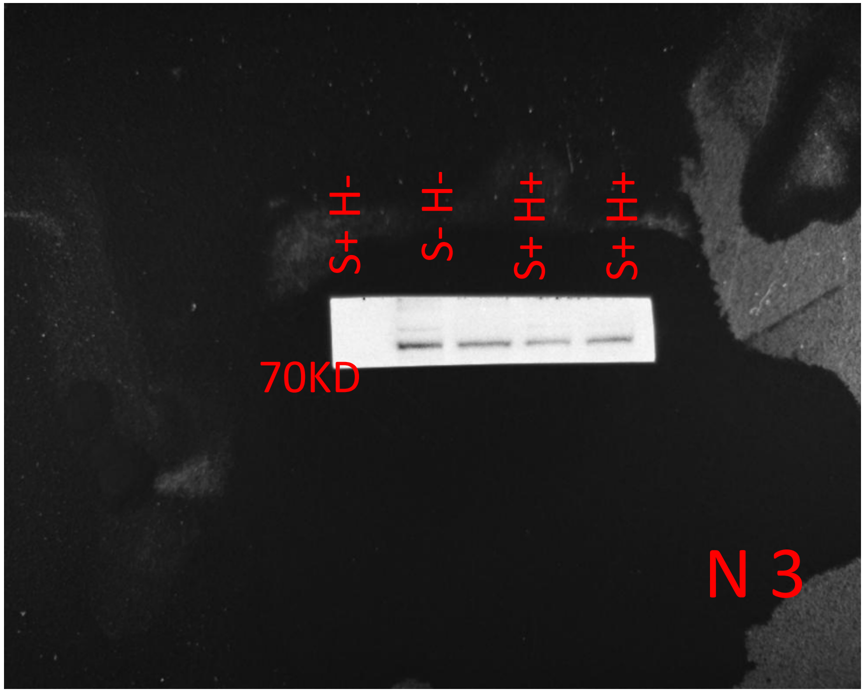

Figure 4. H

S-: without Saracatinib
S+: with Saracatinib
H-： without Hemin
H+: with Hemin

BECN 1

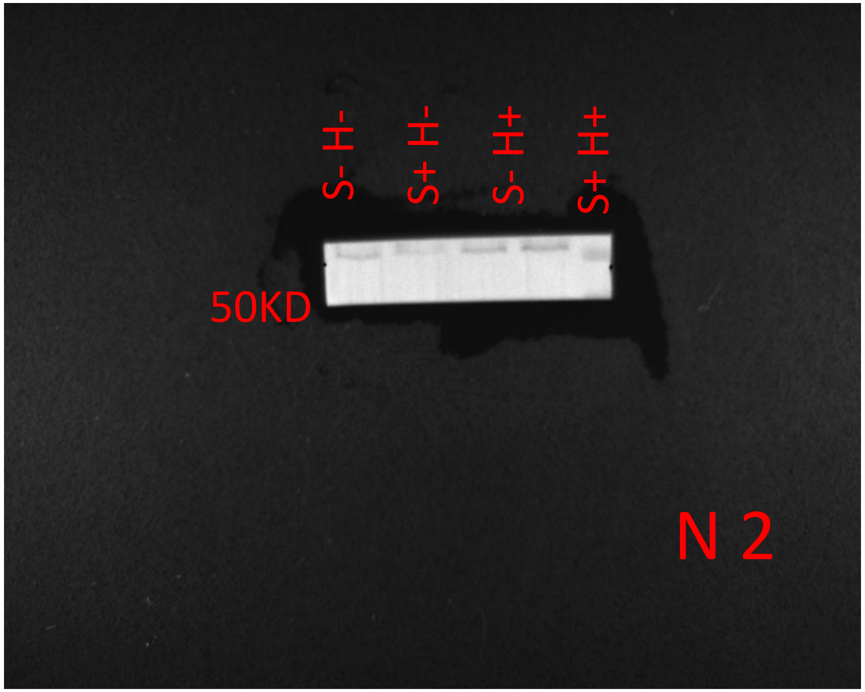

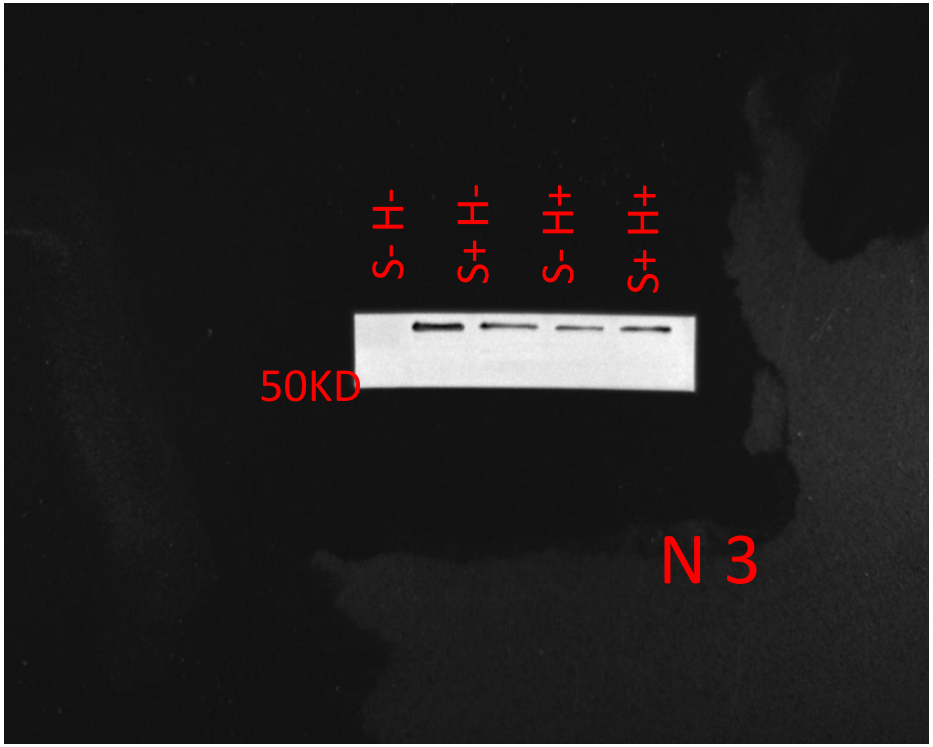

Supplement: Supplementary file 1 — Supplementary file1 (PDF 1209 KB) [file 13577_2024_1034_MOESM1_ESM.pdf]
